# Quinazolinobenzodiazepine Derivatives, Novobenzomalvins A–C: Fibronectin Expression Regulators from *Aspergillus novofumigatus*

**DOI:** 10.3797/scipharm.1106-21

**Published:** 2011-10-03

**Authors:** Kazuki Ishikawa, Tomoo Hosoe, Takeshi Itabashi, Fumiaki Sato, Hiroshi Wachi, Hiromasa Nagase, Takashi Yaguchi, Ken-Ichi Kawai

**Affiliations:** 1 Faculty of Pharmaceutical Sciences, Hoshi University, 2-4-41 Ebara, Shinagawa-ku, Tokyo 142-8501, Japan; 2 Medical Mycology Research Center, Chiba University, 1-8-1 Inohana, Chuo-ku, Chiba 260-8673, Japan

**Keywords:** *Aspergillus novofumigatus*, Novobenzomalvin, Quinazolinobenzodiazepine, Fibronectin expression regulator, Alkaloids, X-ray crystallography

## Abstract

Three new quinazolinobenzodiazepine derivatives, novobenzomalvins A (**1**), B (**2**), and C (**3**), have been isolated as fibronectin expression regulators from *Aspergillus novofumigatus* CBS117520. The structures of **1** to **3** were established by spectroscopic and physicochemical analysis, and chemical investigation including the total synthesis of **1**. Treatment with novo-benzomalvins A (**1**), B (**2**), C (**3**), and *N*-methylnovobenzomalvin A (**5**) increased the expression of fibronectin in normal human neonatal dermal fibroblast cells.

## Introduction

**Fig. 1:**
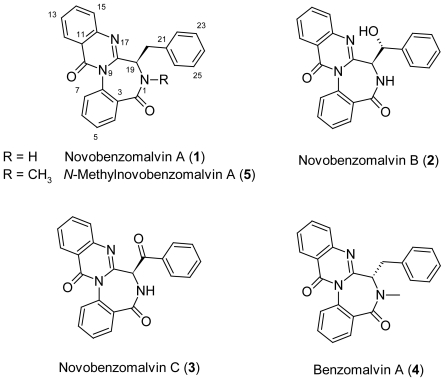
Structures of compounds **1–5**

The fungus *Aspergillus fumigatus* is known as an important human pathogen, which produces many secondary metabolites. Recently, Hong *et al*. re-identified the fungus *Aspergillus novofumigatus* CBS11520 as the new *Aspergillus* sp., which is closely related to *A. fumigatus* [1]. We have previously reported the isolation of two diketopiperazines, novoamauromine and *ent*-cycloechinulin, and a cyclic tripeptide, novofumigatamide, from the MeOH extract of this fungus cultivated on rice [2, 3]. Further investigation of this fungal metabolite led to the isolation of three new quinazolinobenzoziazepine derivatives: novobenzomalvins A (**1**), B (**2**), and C (**3**) (Figure 1). This report describes the isolation, structure, and biological activity of **1**–**3**.

## Results and Discussion

Solid-substrate fermentation cultures of *A. novofumigatus* CBS117520 grown on rice were extracted with MeOH. The evaporated extract was suspended in water and extracted with EtOAc. The evaporated extract was partitioned with MeCN and *n*-hexane to yield a MeCN-soluble fraction. The fraction was extracted sequentially with *n*-hexane, benzene, CHCl_3_, EtOAc, and MeOH. The benzene extract was chromatographed on a Sephadex LH-20 column, followed by medium-pressure liquid column chromatography (MPLC) on silica gel. Thin-layer chromatography (TLC) analysis led to the separation of the compound-containing fractions. Further purification of these fractions by high-performance liquid chromatography (HPLC) yielded **1**, **2**, and **3** along with the known compounds *epi*-aszonalenin A and C, and helvolic acid [4–7].

**Fig. 2:**
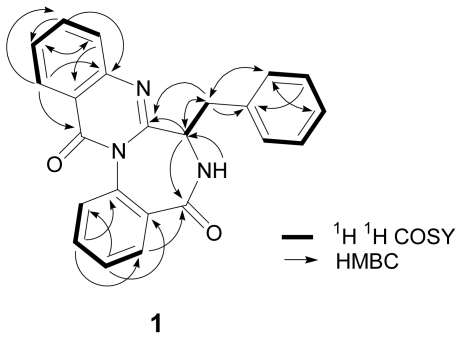
Selected ^1^H ^1^H COSY and HMBC correlations of novobenzomalvin A (**1**)

The molecular formula of **1** was determined to be C_23_H_17_N_3_O_2_ (17 degrees of unsaturation) by high-resolution chemical ionization mass spectrometry (HRCIMS). The presence of NH groups was deduced from the broad absorption at 3437 cm^−1^ in the compound’s IR spectrum. Two carbonyl groups were inferred from its ^13^C NMR spectrum (168.7 and 161.4 ppm) and the absorptions at 1690 and 1665 cm^−1^ in its IR spectrum. The presence of a quinazolinobenzodiazepine ring with an attached benzyl group on C-19 of its ring were deduced from ^1^H, ^13^C NMR, ^1^H ^1^H COSY, and HSQC data (Figure 2).

These results suggested that the planar structure of **1** was a demethylated metabolite of benzomalvin A (**4**), which was originally isolated from *Penicillium* spp [8]. However, the optical rotation [+145° (*c* 0.19, MeOH)] of the *N*-methylated product (**5**) derived from **1** [+136° (*c* 1.09, MeOH)] was clearly different from that of benzomalvin A (**4**) [−106° (*c* 1.0, MeOH)] [8]. Thus, **1** was presumed to have the *R* configuration at C-19. To confirm the stereochemistry of **1**, the compound was synthesized from d-phenylalanine by a modified method [9] (Scheme 1), and the optical rotation of the synthetic product (**1**) was +111° (*c* 0.73, MeOH). From the above results, the chemical structure of **1** was confirmed to be identical to those as shown in Figure 1.

**Fig. 3:**
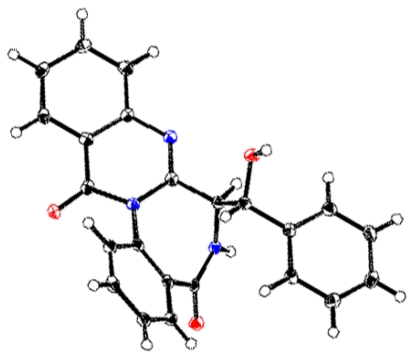
X-ray crystal structure of novobenzomalvin B (**2**) with thermal ellipsoids at 50% probability

The molecular formula of **2** was found to be C_23_H_17_N_3_O_3_ by HREIMS. The ^1^H and ^13^C NMR spectra of **2** was similar to those of **1**, expect for the downfield shift of the carbon at C-20 from δ 35.2 in **1** to δ 72.9 in **2** and the new appearance of a methine signal (δ 5.32) in **2** instead of a methylene signal (δ 3.29 and δ 3.59) in **1** (Table 1). Analysis of the ^1^H ^1^H COSY and HMBC spectra supported the planar structure of **2** being the 20-hydroxy derivative of **1** (Figure 2). X-ray crystallographic analysis of novobenzomalvin B (**2**) was performed to confirm the structure, as the crystal of **2** was suitable for X-ray analysis. The result established the absolute configuration of **2** as shown in Figure 3 [10], in light of the Flack parameter [11] of −0.04(12), using anomalous dispersion of 1263 Friedel pairs [12, 13].

The molecular formula (C_23_H_15_N_3_O_3_) of **3** suggested the loss of two protons from that of **2** (C_23_H_17_N_3_O_3_) and thus indicated the replacement of a hydroxyl group in **2** with a carbonyl group in **3**. The ^1^H and ^13^C NMR spectra of **3** and **2** showed only the difference of a carbonyl sp^2^ carbon signal (δ 188.9) in **3** instead of a secondary hydroxyl group (δ 72.9, δ 5.32) in **2** (Table 1). The analysis of the ^1^H ^1^H COSY, and HMBC spectra supported **3** having a planar structure as the 20-didehydro derivative of **2**, that is, the 20-oxo derivative of **1** (Figure 2). Novobenzomalvin C (**3**) was prepared by treatment of **2** with pyridinium chlorochromate (PCC). The optical rotation of **3** derived from **2** was −329° (*c* 0.14, MeOH), whereas that of naturally occurring **3** was −256° (*c* 0.18, MeOH). Therefore, the absolute structure of **3** was determined to be the one shown in Figure 1.

A wide variety of quinazolinobenzodiazepine alkaloids have been recently reported as natural products from several filamentous fungi, for example: sclerotigenin, an anti-insect active compound isolated from the sclerotia of *Penicillium sclerotigenum* [14]; asperlicins A–E, potent nonpeptidal cholecystokinin antagonists isolated from *Aspergillus alliaceus* [15–17]; benzomalvins, substance-P inhibitors isolated from *Penicillium* sp. [8]; and circumdatins A–I isolated from *Aspergillus ochraceus* [18–21] and *Exphiala* sp. [22]. These alkaloids which have been recently reported as natural products consist of two anthranilic acids and a l-amino acid (*e.g.*, benzomalvin A contains l-phenylalanine, asperlicin C contains l-tryptophan, and circumdatin C, F, G, and I contain l-alanine, etc.) and the all C-19 configuration of them is the same as that of l-amino acids. Interestingly, the C-19 configuration of **1**–**3** is opposite that of other quinazolinobenzodiazepine alkaloids, and this fact suggests that the only *A. novofumigatus* in genus *Aspergillus* utilized d-amino acid in its biosynthesis. Therefore, it might be possible to utilize novobenzomalvins for good chemotaxonomic markers of *Aspergillus* section Fumigati.

**Fig. 4:**
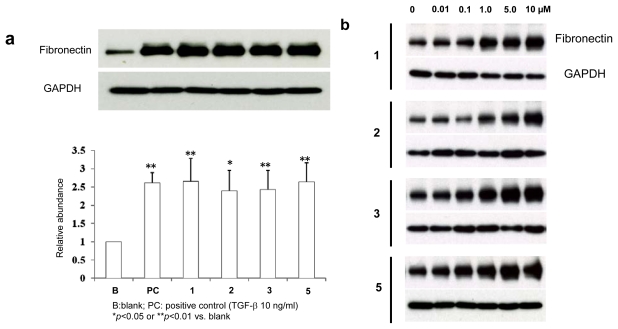
Effect of novobenzomalvins on expression of fibronectin in NHDF-neo cells. **a**: Normal human neonatal dermal fibroblast (NHDF-neo) cells were incubated with novobenzomalvins (each 10 μM), or TGF-β (10 ng/mL) as a positive control for 24 h. **b**: NHDF-neo cells were incubated with 0.01, 0.1, 1.0, 5.0, and 10.0 μM novobenzomalvins for 24 h.

We studied effects of novobenzomalvins A (**1**)–C (**3**) and *N*-methylnovobenzomalvin A (**5**) on the expression of fibronectin (FN). Normal human neonatal dermal fibroblast (NHDF-neo) cells were incubated with novobenzomalvins (each 10 μM), or TGF-β (10 ng/mL) as a positive control for 24 h. Treatments of cells with all of the novobenzomalvins increased the expression of fibronectin, as well as that of TGF-β (10 ng/mL) as a positive control (Figure 4). Fibronectin is an important ingredient of the extracellular matrix proteins in the skin, and is often used as an index for evaluating the extent of matrix accumulation [23]. Although previous reports have shown that some alkaloids such as berberine and cepharanthine effectively reduced fibronectin protein expression [24, 25], little is known about stimulation of fibronectin expression by other alkaloids. In the present study, our data revealed that fibronectin increases in NHDF-neo cultured in the treatment with novobenzomalvins, indicating novobenzomalvins could activate the skin function through stimulating extracellular matrix accumulation. To our knowledge, this is the first reported example that quinazolinobenzodiazepine alkaloids increased the expression of fibronectin.

We would like to examine their functional mechanism in future research. In addition, the antifungal and cytotoxic activities of **1**–**3** were studied using a previously reported method [26]. **1**–**3** showed non-specific antifungal activity against four human pathogenic fungi (*Aspergillus niger*, *A. fumigatus*, *Candida albicans,* and *Cryptococcus neoformans*) tested at 100 μg per disk, and these compounds did not inhibit the cell proliferation in A549, Hela, and LNCap cells at 100 μM.

## Experimental

### General

Melting points were determined on a micro-melting point apparatus (Yanagimoto Ltd., Kyoto, Japan) and are uncorrected. CI and EIMS data were measured on a JMS-MS 600W spectrometer (JEOL Co. Ltd., Tokyo, Japan). UV and IR spectra were recorded on an Ultrospec 2100 pro UV–visible spectrophotometer (Amersham Biosciences Ltd., Tokyo, Japan) and a FT/IR-4100 instrument (JASCO Co. Ltd., Tokyo, Japan), respectively. ^1^H and ^13^C NMR spectra were recorded using a Bruker AVANCE-400 spectrometer (400.13 MHz for ^1^H, 100.61 MHz for ^13^C, Bruker Biospin K. K., Kanagawa, Japan). Chemical shifts (δ) were measured in ppm using tetramethylsilane as an internal standard. CD curves were determined on a J-820 spectropolarimeter (JASCO Co. Ltd.). Optical rotations were measured with a P-1020 photopolarimeter (JASCO Co. Ltd.). TLC was visualized by UV light at 254 nm and/or by spraying with phosphomolybdic acid (5%)/ceric acid (trace) in 5% H_2_SO_4_ and then heating. Column chromatography was performed using a Sephadex LH-20 (GE Healthcare Bio-Science AB, Uppsala, Sweden). MPLC was performed using a Chemco Low-Prep 81-M-2 pump (Chemco Scientific Co. Ltd., Osaka, Japan) and an ULTRA PACK SI-40B column (300 × 26 mm, Yamazen Corp., Osaka, Japan). HPLC was performed using a Senshu SSC-3160 pump (flow rate 7 mL/min, Senshu Scientific Co. Ltd., Tokyo, Japan) and a YMCPack PEGASIL Silica 60-5 column (300 × 10 mm, YMC Co. Ltd., Kyoto, Japan), equipped with a YRD-883 RI detector (Shimamuratech Ltd., Tokyo, Japan). Microwave irradiation reactions were performed in a microwave synthesizer (Initiator 1, Biotage Japan Ltd., Tokyo, Japan).

### Isolation of metabolites from Aspergillus novofumigatus CBS117520

Polished rice (Akitakomachi, 24 kg) was soaked in water for 30 min and then sterilized with an autoclave. *A. novofumigatus* CBS117520 was cultivated for 14 days in Roux flasks, each containing 140 g of moist rice. The cultivated rice was extracted with MeOH, and the extract was concentrated in *vacuo*. The residue was suspended in water and extracted with EtOAc. The EtOAc extract (52 g) was partitioned between *n*-hexane and MeOH to yield a MeCN-soluble mixture. The MeCN extract (29.4 g) was extracted sequentially with *n*-hexane, benzene, CHCl_3_, EtOAc, and MeOH (100 mL each). The benzene extract (18 g) was chromatographed on a Sephadex LH-20 column [solvent system: *n*-hexane/CHCl_3_ (1:4), 180 mL; CHCl_3_/acetone (3:2), 220 mL; CHCl_3_/acetone (1:4), 200 mL; acetone, 200 mL; and then MeOH, 500 mL] to yield five fractions. Fraction 2 [CHCl_3_/acetone (3:2) eluate] was re-chromatographed by MPLC on silica gel [*n*-hexane/acetone (2:1) to acetone] followed by HPLC [CHCl_3_/acetone (15:1)] to give novobenzomalvins A (**1**: 75 mg), B (**2**: 100 mg), and C (**3**: 21 mg), along with *epi*-aszonalenins A and C, and helvolic acid. The spectral data of all compounds were identical to those in the literature [4–7].

#### Novobenzomalvin A ((7R)-7-benzyl-6,7-dihydroquinazolino[3,2-a][1,4]benzodiazepine-5,13-dione, **1**)

Colorless amorphous solid; [α]_D_^20^ +136° (*c* 1.09, MeOH); UV (MeOH) λ_max_ (log ɛ) 213 (4.4), 227 (4.4), 269 (3.7), 279 (3.7), 310 (3.4) nm; IR (KBr) *ν*_max_ 3437, 1690, 1665, 1615, 1597 cm^−1^; CD (0.082 mM, MeOH) λ_max_ (Δɛ) 210 (−33.5), 231(14.9), 254 (−5.2), 287 (1.8) nm. The ^1^H and ^13^C NMR data, see Table 1; HRCIMS m/z [M+H]^+^ 368.1382 (calcd for C_23_H_18_N_3_O_2_, 368.1372).

#### Novobenzomalvin B ((7S)-7-[Hydroxy(phenyl)methyl]-6,7-dihydroquinazolino[3,2-a][1,4]benzodiazepine-5,13-dione, **2**)

Colorless prisms (acetone/MeOH); mp 228–231°C; [α]_D_^20^ + 26° (*c* 0.25, MeOH); UV (MeOH) λ_max_ (log ɛ) 218 (4.7), 228 (4.7), 270 (4.0), 280 (4.0), 311 (3.8), 317 (0.3) nm; IR (KBr) ν_max_ 3420 (br), 1692, 1673, 1614, 1597 cm^−1^; CD (0.052 mM, MeOH) λmax (Δɛ) 210 (−27.8), 232 (14.7), 253 (−4.8), 278 (2.9) nm. The ^1^H and ^13^C NMR data, see Table 1; HMBC (400 MHz, CDCl_3_) 1-NH to C-19, H-4 to C-2, C-3, C-5, H-5 to C-3, H-12 to C-10, C-14, C-16, H-15 to C-11, C-14, C-16, H-19 to C-2, C-18, C-20, C-21, H-20 to C-18, C-19, C-21, C-22, H-22(26) to C-20, H-23(25) to C-21, H-24 to C-22, C-26. EIMS m/z 383[M]^+^ (20), 365 (12), 277 (92), 249 (100), 234 (39), 220 (27), 192 (16), 174 (15), 130 (27), 77 (57); HREIMS m/z [M]^+^ 383.1248 (calcd for C_23_H_17_N_3_O_3_, 383.1270).

#### Novobenzomalvin C ((7S)-7-(Phenylcarbonyl)-6,7-dihydroquinazolino[3,2-a][1,4]benzodiazepine-5,13-dione, **3**)

Colorless amorphous solid; [α] _D_^20^ −256° (*c* 0.18, MeOH); UV(MeOH) λ_max_ (log ɛ) 211 (4.5), 229 (4.5), 281 (3.8), 310 (3.6) nm; IR (KBr) ν_max_ 3437 (br), 1697, 1672, 1615, 1599 cm^−1^; CD (0.052 mM, MeOH) λ_max_ (Δɛ) 212 (0.5), 218 (−0.8), 229 (1.0), 250 (2.2), 296 (−0.3), 317 (0.2) nm. The ^1^H and ^13^C NMR data, see Table 1; HMBC (400 MHz, CDCl_3_) 1-NH to C-19, H-4 to C-2, C-3, C-5, H-5 to C-3, H-12 to C-10, C-14, C-16, H-15 to C-11, C-14, C-16, H-19 to C-2, C-18, C-20, C-21, H-20 to C-18, C-19, C-21, C-22, H-22(26) to C-20, H-23(25) to C-21, H-24 to C-22, C-26. EIMS m/z 381[M]^+^ (73), 105 (100); HREIMS m/z [M]^+^ 381.1094 (calcd for C_23_H_15_N_3_O_3_, 381.1113).

### Methylation of novobenzomalvin A (1)

Methyl iodide (2 mL) was added to a solution of **1** (8 mg) and KOH (10 mg) in DMSO (2 mL) and the mixture was stirred at room temperature (rt) for 1 h. The reaction mixture was added to H_2_O (3 mL) and extracted three times with CHCl_3_. The obtained CHCl_3_ solution was washed several times with H_2_O. After addition of anhydrous Na_2_SO_4_, the filtered solution was evaporated in *vacuo*. The resulting residue (9.1 mg) was purified by HPLC [benzene/acetone (18:1)] to give *N*-methylnovobenzomalvin A (**5**: 3.8 mg).

#### N-Methylnovobenzomalvin A ((7R)-7-Benzyl-6-methyl-6,7-dihydroquinazolino[3,2-a][1,4]benzodiazepine-5,13-dione, **5**)

Colorless amorphous solid; [α]_D_^20^+145° (*c* 0.19, MeOH); UV(MeOH) λ_max_ (log ɛ) 212 (4.4), 229 (4.4), 230 (4.4), 270 (3.9), 309 (3.5) nm; CD (0.079 mM, MeOH) λ_max_ (Δɛ) −77.9 (209), 22.2 (233), −11.6 (255), 8.4 (278), 4.7 (309), 4.0 (322) nm. ^1^H NMR (CDCl_3_, 400 MHz) δ 8.33 (1H, d, J=7.7 Hz), 7.96 (1H, d, J=7.8 Hz),7.84 (2H, d, J = 4.1 Hz), 7.62 (2H, dd, J = 6.3, 1.8 Hz), 7.56 (2H, m), 7.27 (4H, m), 7.22 (1H, m), 4.90 (1H, t, J = 7.7 Hz), 3.82 (1H, dd, J = 14.6, 7.8 Hz), 3.44 (1H, dd, J = 14.6, 6.9 Hz), 3.11 (3H, s). ^13^C NMR (CDCl_3_, 100 MHz) δ 167.3 (C-2), 161.2 (C-10), 151.8 (C-18), 145.95 (C-16), 136.7 (C-21), 134.8 (C-14), 132.9 (C-8), 131.4 (C-6), 130.9 (C-3), 129.9 (C-4), 129.0 (C-22, C-26), 128.95 (C-5), 128.88 (C-7), 128.7 (C-23, C-25), 127.72 (C-15), 127.68 (C-13), 127.6 (C-12), 126.9 (C-24), 121.7 (C-11), 58.3 (C-19), 33.2 (C-20), and 27.9 (N-CH_3_). EIMS m/z 381[M]^+^ (33), 290 (92), 249 (100), 130 (27), 91 (22); HREIMS m/z [M]^+^ 381.1475 (calcd for C_24_H_19_N_3_O_2_ 381.1477).

### Total synthesis of novobenzomalvin A (1)

The total synthesis of novobenzomalvin A (**1**) was accomplished via triflate-catalyzed dehydrative cyclization promoted by microwave irradiation, following the report of Chu et al. (Scheme 1) [8].

#### Methyl 2-[(2-aminobenzoyl)amino]benzoate (**7**)

Anthranilic acid (**6**: 5 g, 0.024 mol) and isatoic acid (8 g, 0.049 mol) was added to H_2_O (80 mL) at rt, and the mixture was stirred at reflux for 2 h. Then, the H_2_O layer was evaporated to give a crude residue. The residue was added to a solution of H_2_SO_4_/MeOH (1:20 v/v, 94.5 mL), and the mixture was stirred at reflux for 2 d. The reaction was quenched by the addition of saturated NaHCO_3_ solution. The aqueous layer was extracted with CHCl_3_. The combined organic layer was evaporated to give a crude residue, which was purified by MPLC [benzene/acetone (4:1)] to afford **7** (3.98 g, 62%) as a colorless amorphous solid. ^1^H-NMR(CDCl_3_): δ 3.87 (3H, s, N-Me), 6.61 (1H, ddd, J = 8.4, 7.3, 1.3, Hz), 6.71 (1H, dd, J = 8.4, 1.3 Hz), 7.06 (1H, dd, J = 8.4, 1.3 Hz), 7.16 (1H, ddd, J = 8.2, 7.3, 1.5 Hz), 7.51 (1H, ddd, J = 8.5, 7.3, 1.3 Hz), 7.59 (1H, dd, J = 8.5, 1.5 Hz), 8.01 (1H, dd, J = 8.2, 1.5 Hz), 8.66 (1H, dd, J = 8.5, 1.3 Hz). ^13^C-NMR (CDCl_3_): δ 52.4, 115.2, 115.6, 116.9, 117.6, 120.4, 122.3, 127.7, 131.0, 132.9, 134.6, 142.0, 149.9, 168.1, 168.9. HREIMS: 270.0979 (M^+^), calcd for C_15_H_14_N_2_O_3_, 270.1004.

#### Methyl 2-({[2-({N-[(benzyloxy)carbonyl]-d-phenylalanyl}amino)phenyl]carbonyl}amino)-benzoate (**8-Z**)

A solution of *N*-[(benzyloxy)carbonyl]-d-phenylalanine (Cbz-d-Phe), 1-ethyl-3-[3-(dimethyl-amino)propyl)]carbodiimide (EDC) (1.8 g, 9.4 mmol), and *N*,*N*-di-*iso*-propyl-ethylamine (DIEA) (2.43 g, 18.8 mmol) in CH_2_Cl_2_ (15 mL) was added dropwise to a solution of **7** (507 mg, 1.88 mmol) in CH_2_Cl_2_ (5 mL) at rt, and the mixture was stirred for 5.5 h at rt. The reaction was quenched by the addition of saturated NaHCO_3_ solution. The aqueous layer was extracted with CHCl_3_. The combined organic layer was evaporated to give a crude residue, which was purified by MPLC [*n*-hexane/CHCl_3_/acetone (2:1:0.4)] to afford **8-Z** (195 mg, 20%) as a colorless amorphous solid. ^1^H NMR (CDCl_3_): δ 3.06 (1H, dd, J = 14.3, 7.0 Hz), 3.14 (1H, dd, J = 14.3, 5.7 Hz), 3.78 (3H, s), 4.56 (1H, dd, J = 13.2, 6.6 Hz), 4.95 (1H, d, J = 12.4 Hz), 5.01 (1H, d, J = 12.4 Hz), 5.58 (1H, m, NH), 6.92-7.00 (2H, m), 7.01-7.10 (6H, m), 7.11-7.23 (4H, m), 7.29 (1H, dd, J = 7.5, 7.4 Hz), 7.36 (1H, dd, J = 7.8, 7.7 Hz), 7.69 (1H, d J = 7.8 Hz), 7.92 (1H, d, J = 8.2 Hz), 8.51-8.61 (2H, m), 11.54 (1H, brs, NH), 11.86 (1H, brs, NH).^13^C NMR (CDCl_3_): δ 38.3, 52.4, 57.2, 66.8, 115.3, 120.4, 120.6, 121.3, 122.9, 123.4, 126.7, 126.9, 127.6 (2C), 127.8, 128.3 (2C), 128.4 (2C), 129.1 (2C), 130.8, 132.8, 134.4, 136.1, 136.2, 139.4, 140.9, 155.7, 167.1, 168.7, 169.7. ESIMS: 574.4 (M+Na)^+^.

#### Methyl 2-({[2-(d-phenylalanylamino)phenyl]carbonyl}amino)benzoate (**8**)

To a solution of **8-Z** (40 mg, 0.073 mmol) in MeOH (2 mL), 20% palladium on carbon (Pd/C) was added at rt, the mixture was stirred for 75 min at rt in a hydrogen atmosphere. The reaction was filtered and concentrated *in vacuo* to give **8** (18.3 mg, 60%). ^1^H-NMR(CDCl_3_): δ 2.82 (1H, dd, J = 13.7, 9.4 Hz), 3.33 (1H, dd, J = 13.7, 4.1 Hz), 3.77 (1H, dd, J = 9.4, 4.1 Hz), 3.95 (3H, s), 7.11-7.32 (8H, m), 7.49-7.64 (3H, m), 7.84 (1H, d, J = 8.0 Hz), 8.09 (1H, d, J = 8.0 Hz), 8.71 (1H, d, J = 8.4 Hz), 8.79 (1H, d, J = 8.6 Hz), 11.85 (1H, brs, NH), 11.91 (1H, brs, NH). ESIMS (%): 440.3 [(M+Na)^+^, 100], 418.3 [(M+H)^+^, 91].

#### Synthetic novobenzomalvin A (**1**)

To a solution of the **8** (18.3 mg) in DMF (0.5 mL) was added tin(II) trifluoromethane sulfonate [Sn(OTf)_2_] (18.3 mg, 0.044 mmol), and the mixture was treated by microwave irradiation at a controlled temperature of 140 °C for 10 min. The reaction was evaporated in vacuo, and was purified by HPLC [*n*-hexane/acetone (2:1)] to afford **1** (13.8 mg, 85%, total yield 6.3%) as a colorless amorphous solid.

### Single-crystal X-ray analysis of novobenzomalvin B (2)

Novobenzomalvin B (**2**) was grown slowly from acetone/MeOH to give colorless prisms. Diffraction intensities were collected from a single crystal having approximate dimensions of 0.51 × 0.16 × 0.10 mm on a Rigaku RAXIS RAPID imaging plate area detector with graphite monochromated Cu-*Kα* radiation. Of 9116 reflections, 2974 were unique reflections. The data were corrected for Lorenz and polarization effects.

#### Crystal data

C_23_H_17_N_3_O_3_, M= 383.41, monoclinic, space group *P*2_1_, a= 10.07473 (18), b= 7.80258 (15), c= 11.3914 (2) Å, β= 104.4659 (12)°, V = 867.07 (3) Å^3^, Z= 2, *D*c= 1.468 g·cm^−3^, *F*(000)= 400, μ(Cu-*Kα*) = 8.109 cm^−1^.

#### Structure solution and refinement

The structure was solved by a direct method using SIR92 [12] and expanded using Fourier techniques with DIRDIF99 [13] and refined by the full-matrix least-squares method. The non-hydrogen atoms were refined anisotropically and hydrogen atoms were refined using the riding model. The final cycle of full-matrix least squares refinement on F^2^ was based on 8714 observed reflections and 280 variable parameters. The final *R* and *Rw* values were 0.0337 and 0.0940, respectively. The absolute structure was deduced based on the Flack parameter [11] −0.04 (12), refined using 1263 Friedel pairs [10]. For more detailed information, see Supporting Information available online.

### Oxidation of novobenzomalvin B (2) with PCC

The CH_2_Cl_2_ solution (0.5 mL) of novobenzomalvin B (**2**: 9 mg) was added to a CH_2_Cl_2_ suspension (1 mL) of PCC (50 mg) and the mixture was refluxed for 29 h with stirring. The reaction mixture was filtered, and the filtrate was evaporated in *vacuo*. The obtained residue was purified by LPLC with CHCl_3_/acetone (15:1) to give novobenzomalvin C (**3**: 2 mg). Synthetic **3** was identical to naturally occurring **3** in comparisons of the ^1^H, ^13^C NMR, [α]_D_^20^, HREIMS, UV, CD, TLC, and HPLC data.

### Immunoblotting

The antibodies used for Western blot analyses including mouse anti-fibronectin antibody (BD Bioscience, Franklin Lakes, NJ, USA) and anti-GAPDH antibody (Millipore, Billerica, MA, USA). Horseradish peroxidase (HRP)-conjugated anti-mouse and -rabbit IgG (GE Healthcare Japan, Tokyo, Japan) were used as secondary antibodies. NHDF-neo cells (Takara Bio Inc., Shiga, Japan) were grown in complete medium: Dulbecco’s modified Eagle’s medium (DMEM) (Sigma, MO, USA) supplemented with 10% (v/v) fetal bovine serum (FBS) (Sigma), l-glutamine and penicillin/streptomycin (Invitrogen Co. Ltd, Carlsbad, CA, USA). Cells were plated at the density of 5 × 10^5^ cells on 24-well plate (TPP, MO, USA). After 24 h incubation with serum-free DMEM, cells were treated with complete medium containing various compounds. At the end of 24 h incubation, cells were washed with cold PBS and lysed on ice in lysis buffer [10 mM Tris-HCl (pH 7.5), 150 mM NaCl, 1 mM EDTA, 1% NP-40, and protease inhibitor cocktail (Roche Applied Science)]. The samples were resolved on 7.5% Tris-Glycine polyacrylamide gels and transferred onto polyvinylidene fluoride (PVDF) membrane (Bio-Rad Bioscience; Hercules, CA). The membranes were blocked for 1 h in TBS containing 0.05% Tween-20 (TBS-T) and 5% fat-free milk and then incubated overnight at 4 °C with the primary antibody. The membranes were washed with TBS-T four times followed by incubation for 1 h at rt with HRP-conjugated anti-mouse IgG and the immunoreactivity was assessed by chemiluminescence.

### Statistical methods

Densitometry analysis was performed using ImageJ 1.43 on immunoblots from three independent experiments. A t-test was performed with SYSTAT software (Hulinks Inc., Tokyo, Japan).

### Antifungal assay

The antifungal assay was performed by using a previously reported method [23]. The antifungal assay was performed using the paper disk method against *A. niger* IFM 41398, *A. fumigatus* IFM 41362, *C. albicans* IFM 40009 and *C. neoformans* ATCC 90112 as test organisms. Novobenzomalvin A (**1**) to C (**3**) were applied to the paper disk (diameter: 8 mm) at 100 μg/disk, and the disks were placed on the assay plates. The test organisms were cultivated in potato dextrose agar (Nissui Pharmaceutical Co., Ltd, Tokyo, Japan) at 25 °C. After 48–72 h of incubation, zones of inhibition (the diameter measured in millimeters) were recorded.

### Cytotoxicity assay

The cytotoxicity assay was performed by a modified method [23]. Cell were seeded into 96-well microplates at 4000 cells/well, allowed to attach for 4–6 h and then incubated in DMEM (Invitrogen Co. Ltd) for A549 human lung cancer cell and Hela human cervical cancer cell, or in RPMI-1640 medium (Wako Pure Chemical Industries, Ltd, Osaka, Japan) supplemented with 10% (v/v) FBS, penicillin G (100 U/mL), streptomycin (100 μg/mL) and amphotericin B (0.25 μg/mL) for LNCap human prostate adenocarcinoma cells until 80% confluence. The media were supplemented with the indicated concentrations of isolated compounds for 48–72 h. Cell proliferation was measured with Cell Counting Kit-8 (Dojindo, Kumamoto, Japan) to count living cells by combining WST-8 (2-(2-methoxy-4-nitrophenyl)-3-(4-nitrophenyl)-5-(2,4-disulfophenyl)-2*H*-tetrazolium) and 1-methoxy PMS (1-methoxy-phenazine methosulfate). Briefly, after the medium was removed, 10 μL of Cell Counting Kit-8 solution was added to each well, and the plates were incubated for 4 h, then cell numbers were obtained by scanning with a Bio-Rad Model Q4 550 microplate reader at 450 nm.

## Supporting Information



## Figures and Tables

**Sch. 1:**
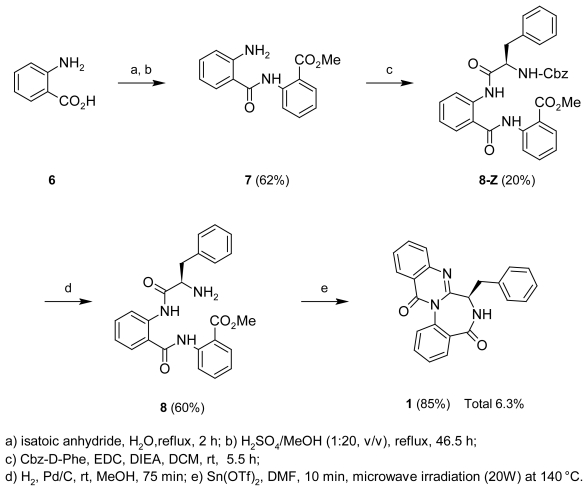
Total synthesis of novobenzomalvin A (**1**)

**Tab. 1:** NMR Spectroscopic Data (400 MHz, CDCl_3_) for Novobenzomalvins A–C

Position	Novobenzomalvin A (1)		Novobenzomalvin B (2)		Novobenzomalvin C (3)	

	δ_C_	δ_H_ (*J* in Hz)	δ_C_	δ_H_ (*J* in Hz)	δ_C_	δ_H_ (*J* in Hz)
1-NH		8.70, d, (5.0)				
2	168.7		167.4		165.7	
3	130.6		132.7		130.9	
4	129.8	7.83, d, (7.5)	130.1	7.92. d (7.7)	130.6	8.04, dd (7.7, 1.5)
5	131.1	7.41, m	131.4	7.61, m	131.7	7.72, ddd (8.1, 7.7, 1.5)
6	128.2	7.54, m	127.0	7.63, m	129.4	7.61, dd (8.1, 7.7)
7	133.2	7.54, m	129.3	7.55, dd (7.9, 7.2)	128.3	7.80, d (8.1)
8	133.2		130.4		133.0	
9-NH						
10	161.4		160.9		161.0	
11	120.8		121.4		121.4	
12	126.9	7.91, d (7.9)	127.6	8.21, d (7.9)	127.4	8.29, dd (8.0, 1.3)
13	127.2	7.26, m	128.3	7.28, m	128.2	7.49, m
14	134.5	7.51, m	135.1	7.75, dd (7.6, 7.5)	134.9	7.67, ddd (8.2, 7.6, 1.3)
15	127.4	7.45, d (8.1)	128.0	4.48, m	127.8	7.41, d, 8.2)
16	145.6		145.0		145.7	
17-NH						
18	154.2	4.35, brdd, (8.5, 5.4)	154.4		152.8	
19	55.9	3.26, dd (14.6, 8.5),	57.5	4.46, brdd (8.0, 5.5)	58.5	5.82, d (5.5)
20	35.2	3.59, dd (14.6, 5.4)	72.9	5.32, dd (6.9, 5.5)	188.9	
21	137.3		139.0		134.6	
22	129.7	7.33, d	126.7	7.45, d (7.6)	128.5	7.87, d (7.9)
23	128.5	7.23, m	128.6	7.33, brt (7.2)	128.9	7.46, dd (7.9, 7.7)
24	126.7	7.18, dd	128.3	7.28, m	134.2	7.59, m
25	128.5	7.23, m	128.6	7.33, brt (7.2)	128.9	7.46, dd (7.9, 7.7)
26	129.7	7.33, d	126.7	7.45, d (7.6)	128.5	7.87, d (7.9)

## References

[1] Hong SB, Go SJ, Shin HD, Frisvad JC, Samson RA (2005). Polyphasic taxonomy of *Aspergillus fumigatus* and related species. Mycologia.

[2] Ishikawa K, Hosoe T, Itabashi T, Wakana D, Takizawa K, Yaguchi T, Kawai K (2010). Novoamauromine and *ent*-Cycloechinulin: Two New Diketopiperazine Derivatives from *Aspergillus novofumigatus*. Chem Pharm Bull.

[3] Ishikawa K, Hosoe T, Itabashi T, Takizawa K, Yaguchi T, Kawai K (2010). A Novofumigatamide, New Cyclic Tripeptide from *Aspergillus novofumigatus*. http://dx.doi.org/10.3987/COM-10-12005.

[4] Rank C, Phipps RK, Harris P, Frisvad JC, Gotfredsen CH, Larsen TO (2006). *epi*-Aszonalenins A, B, and C from *Aspergillus novofumigatus*. Tetrahedron Lett.

[5] Okuda S, Iwasaki S, Tsuda K, Sano Y, Hata T, Udagawa S, Nakayama Y, Yamaguchi H (1964). The Structure of Helvolic Acid. Chem Pharm Bull.

[6] Fujimoto H, Negishi E, Yamaguchi K, Nishi N, Yamazaki M (1996). Isolation of New Tremorgenic Metabolites from an Ascomycete, *Corynascus setosus*. Chem Pharm Bull.

[7] Lee SY, Kinoshita H, Ihara F, Igarashi Y, Nihira T (2008). Identification of novel Derivative of Helvolic Acid from *Metarhizium anisopliae* Grown in Medium with Insect Component. J Biosci Bioeng.

[8] Sun HH, Barrow CJ, Sedlock DM, Gillum AM, Cooper R (1994). Benzomalvins, new substance P inhibitors from a *Penicillium* sp. J Antibiot.

[9] Tseng MC, Lai CY, Chu YW, Chu YH (2009). Tin triflate-mediated total synthesis of circumdatin F, sclerotigenin, asperlicin C, and other quinazolino[3,2-a][1,4]benzodiazepines. Chem Commun.

[b10-scipharm-2011-79-937] 10CCDC 795745 contains the supplementary crystallographic data for this paper. These data can be obtained free of charge via WWW.ccdc.cam.ac.uk/data request/cif, or by mailing data request@ccdc.cam.ac.uk, or by contacting The Cambridge Crystallographic Data Centre, 12, Union Road, Cambridge CB2 1EZ, UK; fax: +44 1223 336033.

[11] Flack HD (1983). On enantiomorph-polarity estimation. Acta Cryst.

[12] Altomare A, Cascarano G, Giacovazzo C, Guagliardi A, Burla M, Polidori G, Camalli M (1994). SIR92 – a program for automatic solution of crystal structures by direct methods. J Appl Cryst.

[13] Beurskens PT, Admiraal G, Beurskens G, Bosman WP, Gelder R, Israel R, Smits JMM (1999). The DIRDIF-99 program system, Technical Report of the Crystallography Laboratory.

[14] Joshi BK, Gloer JB, Wicklow DT, Dowd PF (1999). Sclerotigenin: A New Antiinsectan Benzodiazepine from the Sclerotia of *Penicillium sclerotigenum*. J Nat Prod.

[15] Liesch JM, Hensens OD, Springer JP, Chang RS, Lotti VJ (1985). Asperlicin, A novel non-peptidal cholecystokinin antagonist from *Aspergillus alliaceus*. J Antibiot.

[16] Chang RS, Lotti VJ, Monaghan RL, Birnbaum J, Stapley EO, Goetz MA, Albers-Schonberg G, Patchett AA, Liesch JM, Hensens OD, Springer JP (1985). A potent nonpeptide cholecystokinin antagonist selective for peripheral tissues isolated from *Aspergillus alliaceus*. Science.

[17] Liesch JM, Hensens OD, Zink DL, Goetz MA (1988). Novel cholecystokinin antagonists from *Aspergillus alliaceus* II. Structure determination of asperlicins B, C, D, and E. J. Antibiot.

[18] Rahbæk L, Breinholt J, Frisvad JC, Christophersen C (1999). Circumdatin A, B, and C: Three New Benzodiazepine Alkaloids Isolated from a Culture of the Fungus *Aspergillus ochraceus*. J Org Chem.

[19] Rahbæk L, Breinholt J (1999). Circumdatins D, E, and F: Further Fungal Benzodiazepine Analogues from *Aspergillus ochraceus*. J Nat Prod.

[20] Dai J-R, Carte BK, Sidebottom PJ, Yew ALS, Ng S-B, Huang Y, Butler MS (2001). Circumdatin G, a New Alkaloid from the Fungus *Aspergillus ochraceus*. J Nat Prod.

[21] López-Gresa MP, González MC, Primo J, Moya P, Romero V, Estornell E (2005). Circumdatin H, a new inhibitor of mitochondrial NADH oxidase, from *Aspergillus ochraceus*. J Antibiot.

[22] Zhang D, Yang X, Kang JS, Choi HD, Son BW (2008). Circumdatin I, a New Ultraviolet-A Protecting Benzodiazepine Alkaloid from a Marine Isolate of the Fungus *Exophiala*. J Antibiot.

[23] Schena FP, Gesualdo L (2005). Pathogenetic mechanisms of diabetic nephropathy. J Am Soc Nephrol.

[24] Liu W, Tang F, Deng Y, Li X, Lan T, Zhang X, Huang H, Liu P (2009). Berberine reduces fibronectin and collagen accumulation in rat glomerular messengial cells cultured under high glucose condition. Mol Cell Biochem.

[25] Hayama M, Inoue R, Akiba S, Sato T (2000). Inhibitory effect of cepharanthine on fibronectin production in growth factor-stimulated rat mesangial cells. Eur J Pharmacol.

[26] Wakana D, Hosoe T, Wachi H, Itabashi T, Fukushima K, Yaguchi T, Kawai K (2009). The cytotoxic and antifungal activities of two new sesquiterpenes, malfilanol A and B, derived from *Malbranchea filamentosa*. J Antibiot.

