# Supplementary material for: Quinazolinobenzodiazepine Derivatives, Novobenzomalvins A–C: Fibronectin Expression Regulators from Aspergillus novofumigatus
Source: Sci Pharm. 2011 Oct 3;79(4):937–50. doi: 10.3797/scipharm.1106-21 (PMC3221506; doi:10.3797/scipharm.1106-21)
Supplement: Supplementary file 1 [file scipharm-2011-79-937-s001.pdf]

Scientia Pharmaceutica www.scipharm.at

Open Access

Supporting Information to

Quinazolinobenzodiazepine Derivatives, Novobenzomalvins A–C: Fibronectin Expression Regulators from *Aspergillus novofumigatus*

Kazuki ISHIKAWA, Tomoo HOSOE, Takeshi ITABASHI, Fumiaki SATO, Hiroshi WACHI, Hiromasa NAGASE, Takashi YAGUCHI, Ken-Ichi KAWAI

Published in Sci Pharm. 2011; 79: 937–950 doi:10.3797/scipharm.1106-21

Available from: http://dx.doi.org/10.3797/scipharm.1106-21

© Ishikawa *et al.*; licensee Österreichische Apotheker-Verlagsgesellschaft m. b. H., Vienna, Austria.

This is an Open Access article distributed under the terms of the Creative Commons Attribution License (http://creativecommons.org/licenses/by/3.0/), which permits unrestricted use, distribution, and reproduction in any medium, provided the original work is properly cited.

Table of Contents

X-ray Structure Report (Novobenzomalvin B)

Experimental

Data Collection

A colorless prism crystal of $O_3N_3C_{23}H_{17}$ having approximate dimensions of 0.51 x 0.16 x 0.10 mm was mounted on a glass fiber. All measurements were made on a Rigaku RAXIS RAPID imaging plate area detector with graphite monochromated Cu-Kα radiation.

Indexing was performed from 3 oscillations that were exposed for 60 seconds. The crystal-to-detector distance was 127.40 mm.

Cell constants and an orientation matrix for data collection corresponded to a primitive monoclinic cell with dimensions:

a = 10.07473(18) Å
b = 7.80258(15) Å   β = 104.4659(12)°
c = 11.3914(2) Å
V = 867.07(3) $Å^3$

For Z = 2 and F.W. = 383.41, the calculated density is 1.468 g/cm^3. Based on the systematic absences of:

0k0: k ± 2n

packing considerations, a statistical analysis of intensity distribution, and the successful solution and refinement of the structure, the space group was determined to be:

$P2_1$ (#4)

The data were collected at a temperature of −180 ± 1°C to a maximum 2Θ value of 136.5°. A total of 30 oscillation images were collected. A sweep of data was done using ω scans from 80.0 to 260.0o in 30.0o step, at χ=54.0° and ϕ = 0.0°. The exposure rate was 10.0 [sec./°]. A second sweep was performed using ω scans from 80.0 to 260.0° in 30.0° step, at χ=54.0° and ϕ = 90.0°. The exposure rate was 10.0 [sec./°]. Another sweep was performed using ω scans from 80.0 to 260.0o in 30.0o step, at χ=54.0° and ϕ = 180.0°. The exposure rate was 10.0 [sec./°]. Another sweep was performed using ω scans from 80.0 to 260.0° in 30.0° step, at χ=54.0° and ϕ = 270.0°. The exposure rate was 10.0 [sec./°]. Another sweep was performed using ω scans from 80.0 to 260.0° in 30.0° step, at χ=0.0° and ϕ = 0.0°. The exposure rate was 10.0 [sec./°]. The crystal-to-detector distance was 127.40 mm. Readout was performed in the 0.100 mm pixel mode.

Data Reduction

Of the 9116 reflections that were collected, 2974 were unique (R_{int} = 0.039).

The linear absorption coefficient, μ, for Cu-Kα radiation is 8.109 cm^{-1}. An empirical absorption correction was applied which resulted in transmission factors ranging from 0.781 to 0.922. The data were corrected for Lorentz and polarization effects.

Structure Solution and Refinement

The structure was solved by direct methods [27] and expanded using Fourier techniques [28]. The non-hydrogen atoms were refined anisotropically. Hydrogen atoms were refined using the riding model. The final cycle of full-matrix least-squares refinement [29] on F^2 was based on 8714 observed reflections and 280 variable parameters and converged (largest parameter shift was 0.00 times its esd) with unweighted and weighted agreement factors of:

$$R1 = \Sigma \, ||Fo| - |Fc|| \, / \, \Sigma \, |Fo| = 0.0337$$

$$wR2 = [\Sigma \, (w \, (Fo^2 - Fc^2)^2) / \, \Sigma \, w(Fo^2)^2]^{1/2} = 0.0940$$

The standard deviation of an observation of unit weight [30] was 1.00. A Sheldrick weighting scheme was used. Plots of $\Sigma \, w \, (|Fo| - |Fc|)^2$ versus |Fo|, reflection order in data collection, sin Θ/λ and various classes of indices showed no unusual trends. The maximum and minimum peaks on the final difference Fourier map corresponded to 1.24 and −2.36 $e^-/Å^3$, respectively. The absolute structure was deduced based on Flack parameter, −0.04(12), refined using 1263 Friedel pairs [31].

Neutral atom scattering factors were taken from Cromer and Waber [32]. Anomalous dispersion effects were included in Fcalc [33]; the values for Δf' and Δf" were those of Creagh and McAuley [34]. The values for the mass attenuation coefficients are those of Creagh and Hubbell [35]. All calculations were performed using the CrystalStructure [36, 37] crystallographic software package.

Article published in: Sci Pharm. 2011; 79: 937–950. doi:10.3797/scipharm.1106-21

EXPERIMENTAL DETAILS

A. Crystal Data

Empirical Formula	$O_3N_3C_{23}H_{17}$
Formula Weight	383.41
Crystal Color, Habit	colorless, prism
Crystal Dimensions	0.51 X 0.16 X 0.10 mm
Crystal System	monoclinic
Lattice Type	Primitive
Indexing Images	3 oscillations @ 60.0 seconds
Detector Position	127.40 mm
Pixel Size	0.100 mm
Lattice Parameters	a = 10.07473(18) Å<br>b = 7.80258(15) Å<br>c = 11.3914(2) Å<br>β = 104.4659(12) ^{o}<br>V = 867.07(3) Å^3
Space Group	$P2_1$ (#4)
Z value	2
D_{calc}	1.468 g/cm^3
F_{000}	400.00
μ(CuKα)	8.109 cm^{-1}

B. Intensity Measurements

Diffractometer	Rigaku RAXIS-RAPID
Radiation	CuKα (λ = 1.54187 Å)<br>graphite monochromated
Detector Aperture	460 mm x 256 mm
Data Images	30 exposures
ω oscillation Range (χ=54.0, ϕ=0.0)	80.0 - 260.0^{o}
Exposure Rate	10.0 sec./^{o}
ω oscillation Range (χ=54.0, ϕ=90.0)	80.0 - 260.0^{o}
Exposure Rate	10.0 sec./^{o}
ω oscillation Range (χ=54.0, ϕ=180.0)	80.0 - 260.0^{o}
Exposure Rate	10.0 sec./^{o}
ω oscillation Range (χ=54.0, ϕ=270.0)	80.0 - 260.0^{o}
Exposure Rate	10.0 sec./^{o}
ω oscillation Range (χ=0.0, ϕ=0.0)	80.0 - 260.0^{o}
Exposure Rate	10.0 sec./^{o}
Detector Position	127.40 mm
Pixel Size	0.100 mm
$2\theta_{max}$	136.5^{o}
No. of Reflections Measured	Total: 9116<br>Unique: 2974 (R_{int} = 0.039)<br>Friedel pairs: 1263
Corrections	Lorentz-polarization<br>Absorption<br>(trans. factors: 0.781 - 0.922)

C. Structure Solution and Refinement

Structure Solution	Direct Methods (SIR92)
Refinement	Full-matrix least-squares on F^2
Function Minimized	Σ w $(Fo^2 - Fc^2)^2$
Least Squares Weights	$1/[0.0012Fo^2+1.0000\sigma(Fo^2)]/(4Fo^2)$
$2\theta_{max}$ cutoff	135.0^o
Anomalous Dispersion	All non-hydrogen atoms
No. Observations (I>1.50σ(I))	8714
No. Variables	280
Reflection/Parameter Ratio	31.12
Residuals: R1 (I>2.00σ(I))	0.0337
Residuals: R (I>1.50σ(I))	0.0339
Residuals: wR2 (I>1.50σ(I))	0.0940
Goodness of Fit Indicator	1.002
Flack Parameter	-0.04(12)
Max Shift/Error in Final Cycle	0.000
Maximum peak in Final Diff. Map	1.24 $e^-/Å^3$
Minimum peak in Final Diff. Map	-2.36 $e^-/Å^3$

Tab. S1. Atomic coordinates and B_{iso}/B_{eq}

atom	x	y	z	B_{eq}
O1	0.82724(7)	0.25416(11)	0.69070(6)	1.372(16)
O2	0.48037(7)	0.47828(12)	0.28092(6)	1.493(16)
O3	0.90460(7)	0.95282(11)	0.59196(6)	1.355(16)
N1	0.90636(8)	0.48154(13)	0.60471(7)	1.054(18)
N9	0.66702(9)	0.59464(13)	0.41811(7)	1.090(19)
N17	0.84181(8)	0.74031(13)	0.35053(7)	1.122(18)
C2	0.80891(10)	0.39784(15)	0.64305(9)	1.02(2)
C3	0.67201(10)	0.48266(15)	0.62539(9)	1.04(2)
C4	0.60445(11)	0.46452(16)	0.71866(10)	1.24(2)
C5	0.47309(11)	0.52646(16)	0.70571(10)	1.36(2)
C6	0.40524(11)	0.60276(16)	0.59720(10)	1.46(2)
C7	0.47029(10)	0.62251(16)	0.50454(10)	1.32(2)
C8	0.60446(11)	0.56579(15)	0.51859(9)	1.08(2)
C10	0.58334(11)	0.56609(16)	0.29880(9)	1.19(2)
C11	0.63149(11)	0.65418(15)	0.20392(10)	1.11(2)
C12	0.54872(11)	0.65793(16)	0.08536(9)	1.29(2)
C13	0.59070(11)	0.74725(16)	-0.00303(9)	1.37(2)
C14	0.71626(11)	0.83405(16)	0.02557(9)	1.35(2)
C15	0.79863(11)	0.83221(16)	0.14225(10)	1.34(2)
C16	0.75632(11)	0.74199(16)	0.23295(9)	1.14(2)
C18	0.79651(10)	0.67038(15)	0.43501(9)	1.02(2)
C19	0.89624(11)	0.65768(15)	0.55870(9)	1.12(2)
C20	0.87690(11)	0.79944(16)	0.64912(9)	1.22(2)
C21	0.97437(10)	0.77288(15)	0.77279(9)	1.11(2)
C22	1.10909(11)	0.83175(16)	0.79730(10)	1.46(2)
C23	1.19716(11)	0.80893(17)	0.91123(10)	1.68(2)
C24	1.15202(11)	0.72643(17)	1.00231(10)	1.55(2)
C25	1.01926(11)	0.66596(16)	0.97874(10)	1.50(2)
C26	0.93046(11)	0.68971(16)	0.86490(9)	1.33(2)

$B_{eq} = 8/3\ \pi^2(U_{11}(aa^*)^2 + U_{22}(bb^*)^2 + U_{33}(cc^*)^2 + 2U_{12}(aa^*bb^*)\cos\gamma + 2U_{13}(aa^*cc^*)\cos\beta + 2U_{23}(bb^*cc^*)\cos\alpha)$

Tab. S2. Atomic coordinates and B_{iso} involving hydrogens/B_{eq}

atom	x	y	z	B_{eq}
H1	0.9916	0.4152	0.6137	1.32
H4	0.6498	0.4088	0.7918	1.55
H5	0.4296	0.5170	0.7705	1.56
H6	0.3136	0.6415	0.5865	1.71
H7	0.4231	0.6753	0.4307	1.58
H12	0.4636	0.5988	0.0659	1.51
H13	0.5344	0.7501	-0.0835	1.53
H14	0.7451	0.8947	-0.0360	1.68
H15	0.8835	0.8919	0.1610	1.65
H19	0.9834	0.6807	0.5443	1.25
H20	0.7849	0.7998	0.6560	1.41
H22	1.1409	0.8880	0.7356	1.80
H23	1.2887	0.8498	0.9270	1.95
H24	1.2123	0.7118	1.0804	1.77
H25	0.9884	0.6080	1.0404	1.82
H26	0.8389	0.6488	0.8497	1.58
H27	0.8921	1.0526	0.6355	1.68

$B_{eq} = 8/3\ \pi^2(U_{11}(aa^*)^2 + U_{22}(bb^*)^2 + U_{33}(cc^*)^2 + 2U_{12}(aa^*bb^*)\cos\gamma + 2U_{13}(aa^*cc^*)\cos\beta + 2U_{23}(bb^*cc^*)\cos\alpha)$

Tab. S3. Anisotropic displacement parameters

atom	U_{11}	U_{22}	U_{33}	U_{12}	U_{13}	U_{23}
O1	0.0180(3)	0.0145(4)	0.0191(3)	0.0021(3)	0.0037(3)	0.0027(3)
O2	0.0168(3)	0.0200(5)	0.0184(3)	-0.0055(3)	0.0016(3)	0.0005(3)
O3	0.0237(4)	0.0108(4)	0.0166(3)	-0.0005(3)	0.0045(3)	-0.0001(3)
N1	0.0117(4)	0.0133(5)	0.0144(4)	0.0016(4)	0.0021(3)	0.0001(4)
N9	0.0139(4)	0.0130(5)	0.0149(4)	0.0006(3)	0.0043(3)	0.0007(4)
N17	0.0142(4)	0.0137(5)	0.0147(4)	0.0022(4)	0.0036(3)	0.0001(4)
C2	0.0139(5)	0.0131(6)	0.0095(4)	-0.0023(4)	-0.0014(4)	-0.0031(4)
C3	0.0137(5)	0.0086(6)	0.0161(5)	-0.0025(4)	0.0019(4)	-0.0028(4)
C4	0.0191(5)	0.0122(6)	0.0153(5)	-0.0014(4)	0.0032(4)	-0.0001(4)
C5	0.0182(5)	0.0174(7)	0.0168(5)	-0.0042(5)	0.0055(4)	-0.0031(4)
C6	0.0123(5)	0.0172(6)	0.0255(6)	0.0012(4)	0.0043(4)	-0.0032(5)
C7	0.0153(5)	0.0154(6)	0.0184(5)	-0.0010(4)	0.0022(4)	0.0018(5)
C8	0.0144(5)	0.0126(6)	0.0146(5)	-0.0029(4)	0.0047(4)	-0.0024(4)
C10	0.0154(5)	0.0135(6)	0.0151(5)	0.0029(4)	0.0016(4)	-0.0008(4)
C11	0.0153(5)	0.0111(6)	0.0168(5)	0.0024(4)	0.0055(4)	-0.0013(4)
C12	0.0156(5)	0.0144(6)	0.0177(5)	0.0002(4)	0.0016(4)	-0.0037(4)
C13	0.0193(5)	0.0173(6)	0.0141(5)	0.0050(5)	0.0012(4)	-0.0007(5)
C14	0.0208(5)	0.0174(6)	0.0153(5)	0.0044(5)	0.0084(4)	0.0009(4)
C15	0.0147(5)	0.0161(6)	0.0213(5)	-0.0006(4)	0.0063(4)	-0.0014(5)
C16	0.0170(5)	0.0118(6)	0.0142(5)	0.0028(4)	0.0036(4)	-0.0011(4)
C18	0.0131(5)	0.0092(6)	0.0168(5)	0.0021(4)	0.0044(4)	-0.0025(4)
C19	0.0115(5)	0.0146(6)	0.0162(5)	0.0003(4)	0.0033(4)	0.0013(4)
C20	0.0156(5)	0.0156(6)	0.0159(5)	-0.0006(4)	0.0055(4)	-0.0003(4)
C21	0.0157(5)	0.0106(6)	0.0158(5)	0.0015(4)	0.0037(4)	-0.0031(5)
C22	0.0232(5)	0.0152(7)	0.0181(5)	-0.0019(5)	0.0070(4)	-0.0001(5)
C23	0.0156(5)	0.0192(7)	0.0269(6)	-0.0034(5)	0.0011(4)	-0.0010(5)
C24	0.0237(6)	0.0173(7)	0.0154(5)	0.0040(5)	-0.0001(4)	-0.0029(5)
C25	0.0249(6)	0.0180(7)	0.0155(5)	0.0020(5)	0.0078(4)	-0.0034(5)
C26	0.0142(5)	0.0174(7)	0.0198(5)	-0.0012(4)	0.0056(4)	-0.0035(5)

The general temperature factor expression: $\exp(-2\pi^2(a^{*2}U_{11}h^2 + b^{*2}U_{22}k^2 + c^{*2}U_{33}l^2 + 2a^*b^*U_{12}hk + 2a^*c^*U_{13}hl + 2b^*c^*U_{23}kl))$

Tab. S4. Bond lengths (Å)

atom	atom	distance	atom	atom	distance
O(1)	C(2)	1.2391(14)	O(2)	C(10)	1.2172(13)
O(3)	C(20)	1.4230(14)	N(1)	C(2)	1.3399(14)
N(1)	C(19)	1.4653(15)	N(9)	C(8)	1.4555(14)
N(9)	C(10)	1.4266(11)	N(9)	C(18)	1.4010(13)
N(17)	C(16)	1.3999(11)	N(17)	C(18)	1.2851(14)
C(2)	C(3)	1.4974(14)	C(3)	C(4)	1.4048(16)
C(3)	C(8)	1.3965(13)	C(4)	C(5)	1.3817(15)
C(5)	C(6)	1.3883(15)	C(6)	C(7)	1.3831(17)
C(7)	C(8)	1.3930(15)	C(10)	C(11)	1.4621(17)
C(11)	C(12)	1.3991(13)	C(11)	C(16)	1.3974(15)
C(12)	C(13)	1.3752(16)	C(13)	C(14)	1.3997(15)
C(14)	C(15)	1.3800(13)	C(15)	C(16)	1.4017(17)
C(18)	C(19)	1.5157(12)	C(19)	C(20)	1.5565(16)
C(20)	C(21)	1.5168(12)	C(21)	C(22)	1.3935(14)
C(21)	C(26)	1.3963(16)	C(22)	C(23)	1.3885(14)
C(23)	C(24)	1.3905(17)	C(24)	C(25)	1.3799(15)
C(25)	C(26)	1.3917(13)			

Tab. S5. Bond lengths involving hydrogens (Å)

atom	atom	distance	atom	atom	distance
O(3)	H(27)	0.948	N(1)	H(1)	0.986
C(4)	H(4)	0.950	C(5)	H(5)	0.950
C(6)	H(6)	0.950	C(7)	H(7)	0.950
C(12)	H(12)	0.950	C(13)	H(13)	0.950
C(14)	H(14)	0.950	C(15)	H(15)	0.950
C(19)	H(19)	0.950	C(20)	H(20)	0.950
C(22)	H(22)	0.950	C(23)	H(23)	0.950
C(24)	H(24)	0.950	C(25)	H(25)	0.950
C(26)	H(26)	0.950			

Tab. S6. Bond angles (°)

atom	atom	atom	angle	atom	atom	atom	angle
C(2)	N(1)	C(19)	125.63(9)	C(8)	N(9)	C(10)	117.14(8)
C(8)	N(9)	C(18)	121.90(7)	C(10)	N(9)	C(18)	120.34(9)
C(16)	N(17)	C(18)	118.15(8)	O(1)	C(2)	N(1)	122.86(9)
O(1)	C(2)	C(3)	119.35(9)	N(1)	C(2)	C(3)	117.78(9)
C(2)	C(3)	C(4)	117.25(9)	C(2)	C(3)	C(8)	123.67(10)
C(4)	C(3)	C(8)	118.88(9)	C(3)	C(4)	C(5)	121.07(9)
C(4)	C(5)	C(6)	119.37(11)	C(5)	C(6)	C(7)	120.36(10)
C(6)	C(7)	C(8)	120.54(9)	N(9)	C(8)	C(3)	122.98(9)
N(9)	C(8)	C(7)	117.32(8)	C(3)	C(8)	C(7)	119.69(10)
O(2)	C(10)	N(9)	121.56(10)	O(2)	C(10)	C(11)	124.55(8)
N(9)	C(10)	C(11)	113.87(9)	C(10)	C(11)	C(12)	119.71(9)
C(10)	C(11)	C(16)	120.05(9)	C(12)	C(11)	C(16)	120.15(10)
C(11)	C(12)	C(13)	119.95(10)	C(12)	C(13)	C(14)	120.01(8)
C(13)	C(14)	C(15)	120.68(10)	C(14)	C(15)	C(16)	119.65(10)
N(17)	C(16)	C(11)	121.61(10)	N(17)	C(16)	C(15)	118.83(9)
C(11)	C(16)	C(15)	119.55(9)	N(9)	C(18)	N(17)	124.67(8)
N(9)	C(18)	C(19)	118.58(9)	N(17)	C(18)	C(19)	116.55(8)
N(1)	C(19)	C(18)	111.38(8)	N(1)	C(19)	C(20)	116.03(8)
C(18)	C(19)	C(20)	113.90(8)	O(3)	C(20)	C(19)	102.92(8)
O(3)	C(20)	C(21)	112.61(8)	C(19)	C(20)	C(21)	111.01(9)
C(20)	C(21)	C(22)	120.88(10)	C(20)	C(21)	C(26)	120.55(9)
C(22)	C(21)	C(26)	118.57(8)	C(21)	C(22)	C(23)	120.53(11)
C(22)	C(23)	C(24)	120.33(10)	C(23)	C(24)	C(25)	119.66(9)
C(24)	C(25)	C(26)	120.13(11)	C(21)	C(26)	C(25)	120.76(10)

Tab. S7. Bond angles involving hydrogens (°)

atom	atom	atom	angle	atom	atom	atom	angle
C(20)	O(3)	H(27)	112.6	C(2)	N(1)	H(1)	113.6
C(19)	N(1)	H(1)	120.7	C(3)	C(4)	H(4)	119.5
C(5)	C(4)	H(4)	119.5	C(4)	C(5)	H(5)	120.3
C(6)	C(5)	H(5)	120.3	C(5)	C(6)	H(6)	119.8
C(7)	C(6)	H(6)	119.8	C(6)	C(7)	H(7)	119.7
C(8)	C(7)	H(7)	119.7	C(11)	C(12)	H(12)	120.0
C(13)	C(12)	H(12)	120.0	C(12)	C(13)	H(13)	120.0
C(14)	C(13)	H(13)	120.0	C(13)	C(14)	H(14)	119.7
C(15)	C(14)	H(14)	119.7	C(14)	C(15)	H(15)	120.2
C(16)	C(15)	H(15)	120.2	N(1)	C(19)	H(19)	104.7
C(18)	C(19)	H(19)	104.7	C(20)	C(19)	H(19)	104.7
O(3)	C(20)	H(20)	110.0	C(19)	C(20)	H(20)	110.0
C(21)	C(20)	H(20)	110.0	C(21)	C(22)	H(22)	119.7
C(23)	C(22)	H(22)	119.7	C(22)	C(23)	H(23)	119.8
C(24)	C(23)	H(23)	119.8	C(23)	C(24)	H(24)	120.2
C(25)	C(24)	H(24)	120.2	C(24)	C(25)	H(25)	119.9
C(26)	C(25)	H(25)	119.9	C(21)	C(26)	H(26)	119.6
C(25)	C(26)	H(26)	119.6				

Tab. S8. Torsion Angles (°)

atom1	atom2	atom3	atom4	angle	atom1	atom2	atom3	atom4	angle
C(2)	N(1)	C(19)	C(18)	-70.95(12)	C(2)	N(1)	C(19)	C(20)	61.54(12)
C(19)	N(1)	C(2)	O(1)	-173.66(8)	C(19)	N(1)	C(2)	C(3)	7.12(14)
C(8)	N(9)	C(10)	O(2)	-19.98(16)	C(8)	N(9)	C(10)	C(11)	158.71(10)
C(10)	N(9)	C(8)	C(3)	137.75(11)	C(10)	N(9)	C(8)	C(7)	-41.22(14)
C(8)	N(9)	C(18)	N(17)	-160.58(11)	C(8)	N(9)	C(18)	C(19)	24.70(15)
C(18)	N(9)	C(8)	C(3)	-51.23(15)	C(18)	N(9)	C(8)	C(7)	129.80(11)
C(10)	N(9)	C(18)	N(17)	10.16(17)	C(10)	N(9)	C(18)	C(19)	-164.56(10)
C(18)	N(9)	C(10)	O(2)	168.85(11)	C(18)	N(9)	C(10)	C(11)	-12.46(15)
C(16)	N(17)	C(18)	N(9)	0.17(17)	C(16)	N(17)	C(18)	C(19)	175.00(10)
C(18)	N(17)	C(16)	C(11)	-7.10(17)	C(18)	N(17)	C(16)	C(15)	173.84(11)
O(1)	C(2)	C(3)	C(4)	39.30(14)	O(1)	C(2)	C(3)	C(8)	-135.45(11)
N(1)	C(2)	C(3)	C(4)	-141.45(10)	N(1)	C(2)	C(3)	C(8)	43.80(14)
C(2)	C(3)	C(4)	C(5)	-174.63(10)	C(2)	C(3)	C(8)	N(9)	-6.90(17)
C(2)	C(3)	C(8)	C(7)	172.04(10)	C(4)	C(3)	C(8)	N(9)	178.43(10)
C(4)	C(3)	C(8)	C(7)	-2.63(16)	C(8)	C(3)	C(4)	C(5)	0.38(17)
C(3)	C(4)	C(5)	C(6)	2.15(17)	C(4)	C(5)	C(6)	C(7)	-2.44(18)
C(5)	C(6)	C(7)	C(8)	0.18(18)	C(6)	C(7)	C(8)	N(9)	-178.62(10)
C(6)	C(7)	C(8)	C(3)	2.37(17)	O(2)	C(10)	C(11)	C(12)	7.99(18)
O(2)	C(10)	C(11)	C(16)	-175.44(11)	N(9)	C(10)	C(11)	C(12)	-170.65(10)
N(9)	C(10)	C(11)	C(16)	5.92(16)	C(10)	C(11)	C(12)	C(13)	177.01(11)
C(10)	C(11)	C(16)	N(17)	3.71(17)	C(10)	C(11)	C(16)	C(15)	-177.23(11)
C(12)	C(11)	C(16)	N(17)	-179.74(11)	C(12)	C(11)	C(16)	C(15)	-0.68(18)
C(16)	C(11)	C(12)	C(13)	0.45(18)	C(11)	C(12)	C(13)	C(14)	0.12(18)
C(12)	C(13)	C(14)	C(15)	-0.46(18)	C(13)	C(14)	C(15)	C(16)	0.22(18)
C(14)	C(15)	C(16)	N(17)	179.43(11)	C(14)	C(15)	C(16)	C(11)	0.35(18)
N(9)	C(18)	C(19)	N(1)	48.51(13)	N(9)	C(18)	C(19)	C(20)	-85.04(12)
N(17)	C(18)	C(19)	N(1)	-126.64(11)	N(17)	C(18)	C(19)	C(20)	99.81(12)
N(1)	C(19)	C(20)	O(3)	166.13(7)	N(1)	C(19)	C(20)	C(21)	45.42(12)
C(18)	C(19)	C(20)	O(3)	-62.55(11)	C(18)	C(19)	C(20)	C(21)	176.74(9)
O(3)	C(20)	C(21)	C(22)	-31.52(15)	O(3)	C(20)	C(21)	C(26)	148.04(10)
C(19)	C(20)	C(21)	C(22)	83.27(13)	C(19)	C(20)	C(21)	C(26)	-97.17(12)
C(20)	C(21)	C(22)	C(23)	179.15(11)	C(20)	C(21)	C(26)	C(25)	-179.61(11)
C(22)	C(21)	C(26)	C(25)	-0.04(14)	C(26)	C(21)	C(22)	C(23)	-0.42(18)
C(21)	C(22)	C(23)	C(24)	0.20(19)	C(22)	C(23)	C(24)	C(25)	0.49(19)
C(23)	C(24)	C(25)	C(26)	-0.94(19)	C(24)	C(25)	C(26)	C(21)	0.72(18)

The sign is positive if when looking from atom 2 to atom 3 a clock-wise motion of atom 1 would superimpose it on atom 4.

Tab. S9. Distances beyond the asymmetric unit out to 3.60 Å

atom	atom	distance	atom	atom	distance
O(1)	O(3)[1]	2.7998(11)	O(1)	N(1)	2.2655(12)
O(1)	N(17)[2]	3.4831(11)	O(1)	C(3)	2.3657(13)
O(1)	C(4)	2.8619(14)	O(1)	C(7)[3]	3.4141(11)
O(1)	C(8)	3.5490(12)	O(1)	C(24)[4]	3.4576(13)
O(2)	N(9)	2.3096(10)	O(2)	C(5)[3]	3.5547(15)
O(2)	C(6)[3]	3.3229(14)	O(2)	C(7)	2.8098(14)
O(2)	C(8)	2.7691(11)	O(2)	C(11)	2.3745(14)
O(2)	C(12)	2.8580(14)	O(2)	C(13)[5]	3.5567(13)
O(2)	C(18)	3.5608(11)	O(3)	O(1)[6]	2.7998(11)
O(3)	N(1)[7]	3.2906(11)	O(3)	N(17)	3.1375(11)
O(3)	N(17)[7]	3.3395(11)	O(3)	C(6)[8]	3.5207(12)
O(3)	C(18)	2.8751(13)	O(3)	C(18)[7]	3.5363(13)
O(3)	C(19)	2.3319(14)	O(3)	C(19)[7]	3.3536(14)
O(3)	C(21)	2.4464(12)	O(3)	C(22)	2.8637(11)
N(1)	O(1)	2.2655(12)	N(1)	O(3)[2]	3.2906(11)
N(1)	N(9)	2.9241(10)	N(1)	N(17)	3.4558(12)
3.0973(12)		N(1)	C(3)	2.4306(13)	
N(1)	C(4)	3.5954(15)	N(1)	C(8)	3.0257(13)
N(1)	C(18)	2.4625(13)	N(1)	C(20)	2.5635(16)
N(1)	C(21)	2.9407(14)	N(1)	C(26)	3.3350(13)
N(9)	O(2)	2.3096(10)	N(9)	N(1)	2.9241(10)
N(9)	N(17)	2.3797(13)	N(9)	C(2)	3.0228(12)
N(9)	C(3)	2.5064(13)	N(9)	C(7)	2.4331(15)
N(9)	C(11)	2.4209(13)	N(9)	C(16)	2.7437(14)
N(9)	C(19)	2.5084(12)	N(9)	C(20)	3.3421(12)
N(17)	O(1)[7]	3.4831(11)	N(17)	O(3)	3.1375(11)
N(17)	O(3)[2]	3.3395(11)	N(17)	N(1)	3.4558(12)
N(17)	N(1)[7]	3.0973(12)	N(17)	N(9)	2.3797(13)
N(17)	C(10)	2.8652(14)	N(17)	C(11)	2.4419(12)
N(17)	C(15)	2.4118(13)	N(17)	C(19)	2.3854(12)
N(17)	C(20)	3.3625(13)	C(2)	N(9)	3.0228(12)
C(2)	C(4)	2.4783(16)	C(2)	C(8)	2.5517(14)
C(2)	C(18)	3.1632(15)	C(2)	C(19)	2.4960(16)
C(2)	C(20)	3.2046(17)	C(2)	C(21)	3.5065(15)
C(2)	C(26)	3.3950(15)	C(3)	O(1)	2.3657(13)
C(3)	N(1)	2.4306(13)	C(3)	N(9)	2.5064(13)
C(3)	C(5)	2.4262(16)	C(3)	C(6)	2.7881(15)

Tab. S9. (Cont.)

atom	atom	distance	atom	atom	distance
C(3)	C(7)	2.4121(13)	C(3)	C(7)[3)]	3.3290(16)
C(3)	C(18)	3.1274(16)	C(3)	C(19)	2.8988(16)
C(3)	C(20)	3.1882(16)	C(4)	O(1)	2.8619(14)
C(4)	N(1)	3.5954(15)	C(4)	C(2)	2.4783(16)
C(4)	C(6)	2.3913(14)	C(4)	C(7)	2.7656(15)
C(4)	C(8)	2.4122(15)	C(5)	O(2)[8)]	3.5547(15)
C(5)	C(3)	2.4262(16)	C(5)	C(7)	2.4045(16)
C(5)	C(8)	2.7926(17)	C(5)	C(11)[3)]	3.3391(17)
C(5)	C(16)[3)]	3.4005(17)	C(6)	O(2)[8)]	3.3229(14)
C(6)	O(3)[3)]	3.5207(12)	C(6)	C(3)	2.7881(15)
C(6)	C(4)	2.3913(14)	C(6)	C(8)	2.4107(17)
C(7)	O(1)[8)]	3.4141(11)	C(7)	O(2)	2.8098(14)
C(7)	N(9)	2.4331(15)	C(7)	C(3)	2.4121(13)
C(7)	C(3)[8)]	3.3290(16)	C(7)	C(4)	2.7656(15)
C(7)	C(5)	2.4045(16)	C(7)	C(8)[8)]	3.5356(16)
C(7)	C(10)	2.8840(16)	C(7)	C(18)	3.5873(15)
C(8)	O(1)	3.5490(12)	C(8)	O(2)	2.7691(11)
C(8)	N(1)	3.0257(13)	C(8)	C(2)	2.5517(14)
C(8)	C(4)	2.4122(15)	C(8)	C(5)	2.7926(17)
C(8)	C(6)	2.4107(17)	C(8)	C(7)[3)]	3.5356(16)
C(8)	C(10)	2.4592(14)	C(8)	C(18)	2.4973(16)
C(8)	C(19)	2.9484(15)	C(8)	C(20)	3.3210(15)
C(10)	N(17)	2.8652(14)	C(10)	C(7)	2.8840(16)
C(10)	C(8)	2.4592(14)	C(10)	C(12)	2.4745(14)
C(10)	C(16)	2.4772(17)	C(10)	C(18)	2.4530(13)
C(11)	O(2)	2.3745(14)	C(11)	N(9)	2.4209(13)
C(11)	N(17)	2.4419(12)	C(11)	C(5)[8)]	3.3391(17)
C(11)	C(13)	2.4020(15)	C(11)	C(14)	2.7737(16)
C(11)	C(15)	2.4186(17)	C(11)	C(18)	2.7428(13)
C(12)	O(2)	2.8580(14)	C(12)	C(10)	2.4745(14)
C(12)	C(13)[5)]	3.5302(17)	C(12)	C(14)	2.4033(17)
C(12)	C(15)	2.7916(15)	C(12)	C(16)	2.4237(13)
C(13)	O(2)[9)]	3.5567(13)	C(13)	C(11)	2.4020(15)
C(13)	C(12)[9)]	3.5302(17)	C(13)	C(15)	2.4155(13)
C(13)	C(16)	2.7876(13)	C(14)	C(11)	2.7737(16)
C(14)	C(12)	2.4033(17)	C(14)	C(16)	2.4048(14)
C(14)	C(24)[7)]	3.3832(17)	C(14)	C(25)[10)]	3.4838(16)

Tab. S9. (Cont.)

atom	atom	distance	atom	atom	distance
C(14)	C(26)[10)]	3.3539(17)	C(15)	N(17)	2.4118(13)
C(15)	C(11)	2.4186(17)	C(15)	C(12)	2.7916(15)
C(15)	C(13)	2.4155(13)	C(15)	C(18)	3.5710(15)
C(15)	C(24)[7)]	3.5818(18)	C(15)	C(25)[10)]	3.4873(17)
C(16)	N(9)	2.7437(14)	C(16)	C(5)[8)]	3.4005(17)
C(16)	C(10)	2.4772(17)	C(16)	C(12)	2.4237(13)
C(16)	C(13)	2.7876(13)	C(16)	C(14)	2.4048(14)
C(16)	C(18)	2.3040(14)	C(16)	C(22)[2)]	3.5266(17)
C(18)	O(2)	3.5608(11)	C(18)	O(3)	2.8751(13)
C(18)	O(3)[2)]	3.5363(13)	C(18)	N(1)	2.4625(13)
C(18)	C(2)	3.1632(15)	C(18)	C(3)	3.1274(16)
C(18)	C(7)	3.5873(15)	C(18)	C(8)	2.4973(16)
C(18)	C(10)	2.4530(13)	C(18)	C(11)	2.7428(13)
C(18)	C(15)	3.5710(15)	C(18)	C(16)	2.3040(14)
C(18)	C(20)	2.5752(14)	C(19)	O(3)	2.3319(14)
C(19)	O(3)[2)]	3.3536(14)	C(19)	N(9)	2.5084(12)
C(19)	N(17)	2.3854(12)	C(19)	C(2)	2.4960(16)
C(19)	C(3)	2.8988(16)	C(19)	C(8)	2.9484(15)
C(19)	C(21)	2.5330(14)	C(19)	C(22)	3.3043(14)
C(19)	C(26)	3.4274(14)	C(20)	N(1)	2.5635(16)
C(20)	N(9)	3.3421(12)	C(20)	N(17)	3.3625(13)
C(20)	C(2)	3.2046(17)	C(20)	C(3)	3.1882(16)
C(20)	C(8)	3.3210(15)	C(20)	C(18)	2.5752(14)
C(20)	C(22)	2.5322(13)	C(20)	C(26)	2.5305(14)
C(21)	O(3)	2.4464(12)	C(21)	N(1)	2.9407(14)
C(21)	C(2)	3.5065(15)	C(21)	C(19)	2.5330(14)
C(21)	C(23)	2.4157(13)	C(21)	C(24)	2.7987(13)
C(21)	C(25)	2.4237(15)	C(22)	O(3)	2.8637(11)
C(22)	C(16)[7)]	3.5266(17)	C(22)	C(19)	3.3043(14)
C(22)	C(20)	2.5322(13)	C(22)	C(24)	2.4107(16)
C(22)	C(25)	2.7747(17)	C(22)	C(26)	2.3984(17)
C(23)	C(21)	2.4157(13)	C(23)	C(25)	2.3952(17)
C(23)	C(26)	2.7668(15)	C(24)	O(1)[11)]	3.4576(13)
C(24)	C(14)[2)]	3.3832(17)	C(24)	C(15)[2)]	3.5818(18)
C(24)	C(21)	2.7987(13)	C(24)	C(22)	2.4107(16)
C(24)	C(26)	2.4019(13)	C(25)	C(14)[12)]	3.4838(16)
C(25)	C(15)[12)]	3.4873(17)	C(25)	C(21)	2.4237(15)

Tab. S9. (Cont.)

atom	atom	distance	atom	atom	distance
C(25)	C(22)	2.7747(17)	C(25)	C(23)	2.3952(17)
C(26)	N(1)	3.3350(13)	C(26)	C(2)	3.3950(15)
C(26)	C(14)[12)]	3.3539(17)	C(26)	C(19)	3.4274(14)
C(26)	C(20)	2.5305(14)	C(26)	C(22)	2.3984(17)
C(26)	C(23)	2.7668(15)	C(26)	C(24)	2.4019(13)

Symmetry Operators:

(1) X,Y-1,Z
(2) -X+2,Y+1/2-1,-Z+1
(3) -X+1,Y+1/2-1,-Z+1
(4) -X+2,Y+1/2-1,-Z+2
(5) -X+1,Y+1/2-1,-Z
(6) X,Y+1,Z
(7) -X+2,Y+1/2,-Z+1
(8) -X+1,Y+1/2,-Z+1
(9) -X+1,Y+1/2,-Z
(10) X,Y,Z-1
(11) -X+2,Y+1/2,-Z+2
(12) X,Y,Z+1

Tab. S10. Distances beyond the asymmetric unit out to 3.60 Å involving hydrogens

atom	atom	distance	atom	atom	distance
O(1)	H(1)	2.411	O(1)	H(4)	2.647
O(1)	H(6)$^{1)}$	3.243	O(1)	H(7)$^{1)}$	2.629
O(1)	H(15)$^{2)}$	3.168	O(1)	H(20)$^{3)}$	3.581
O(1)	H(24)$^{4)}$	2.754	O(1)	H(25)$^{4)}$	3.361
O(1)	H(26)	3.559	O(1)	H(27)$^{3)}$	1.871
O(2)	H(6)$^{1)}$	3.454	O(2)	H(7)	2.470
O(2)	H(12)	2.589	O(2)	H(13)$^{5)}$	2.843
O(2)	H(14)$^{5)}$	3.194	O(2)	H(20)$^{1)}$	3.249
O(2)	H(24)$^{6)}$	3.571	O(3)	H(1)$^{7)}$	2.809
O(3)	H(6)$^{8)}$	2.983	O(3)	H(19)	2.377
O(3)	H(19)$^{7)}$	2.778	O(3)	H(20)	1.963
O(3)	H(22)	2.580	N(1)	H(15)$^{2)}$	3.045
N(1)	H(19)	1.938	N(1)	H(19)$^{2)}$	3.251
N(1)	H(20)	2.892	N(1)	H(26)	3.301
N(1)	H(27)$^{3)}$	3.372	N(9)	H(6)$^{1)}$	3.542
N(9)	H(7)	2.575	N(9)	H(19)	3.223
N(9)	H(20)	3.116	N(9)	H(22)$^{2)}$	3.332
N(17)	H(1)$^{7)}$	2.122	N(17)	H(5)$^{8)}$	3.486
N(17)	H(15)	2.586	N(17)	H(19)	2.356
N(17)	H(22)$^{2)}$	2.938	N(17)	H(27)$^{2)}$	3.024
C(2)	H(1)	1.955	C(2)	H(4)	2.609
C(2)	H(6)$^{1)}$	3.279	C(2)	H(7)$^{1)}$	2.864
C(2)	H(15)$^{2)}$	3.336	C(2)	H(19)	3.196
C(2)	H(20)	3.152	C(2)	H(24)$^{4)}$	3.523
C(2)	H(26)	3.018	C(2)	H(27)$^{3)}$	2.829
C(3)	H(1)	3.298	C(3)	H(4)	2.047
C(3)	H(5)	3.284	C(3)	H(7)	3.269
C(3)	H(7)$^{1)}$	2.603	C(3)	H(20)	2.709
C(3)	H(26)	2.982	C(4)	H(5)	2.034
C(4)	H(6)	3.249	C(4)	H(7)$^{1)}$	2.798
C(4)	H(13)$^{9)}$	3.367	C(4)	H(20)	3.362
C(4)	H(24)$^{4)}$	3.229	C(4)	H(26)	2.849
C(5)	H(4)	2.026	C(5)	H(6)	2.035
C(5)	H(7)	3.260	C(5)	H(7)$^{1)}$	3.439
C(5)	H(13)$^{9)}$	2.907	C(6)	H(4)	3.248
C(6)	H(5)	2.040	C(6)	H(7)	2.030
C(6)	H(27)$^{1)}$	3.491	C(7)	H(5)	3.262

Tab. S10. (Cont.)

atom	atom	distance	atom	atom	distance
C(7)	H(6)	2.030	C(7)	H(20)	3.493
C(8)	H(4)	3.269	C(8)	H(6)	3.266
C(8)	H(7)	2.038	C(8)	H(7)[1)]	3.126
C(8)	H(20)	2.768	C(10)	H(4)[8)]	3.538
C(10)	H(7)	2.608	C(10)	H(12)	2.638
C(10)	H(13)[5)]	3.471	C(10)	H(22)[2)]	3.216
C(10)	H(23)[2)]	3.575	C(11)	H(4)[8)]	3.471
C(11)	H(5)[8)]	2.927	C(11)	H(12)	2.047
C(11)	H(13)	3.261	C(11)	H(15)	3.278
C(11)	H(22)[2)]	3.042	C(11)	H(23)[2)]	3.018
C(12)	H(4)[8)]	3.345	C(12)	H(5)[8)]	3.227
C(12)	H(13)	2.025	C(12)	H(13)[5)]	3.289
C(12)	H(14)	3.257	C(12)	H(14)[5)]	3.531
C(12)	H(23)[6)]	3.162	C(12)	H(23)[2)]	2.932
C(12)	H(24)[6)]	3.402	C(13)	H(5)[10)]	3.229
C(13)	H(5)[8)]	3.429	C(13)	H(12)	2.025
C(13)	H(12)[11)]	2.853	C(13)	H(14)	2.044
C(13)	H(15)	3.273	C(13)	H(23)[6)]	3.053
C(13)	H(23)[2)]	3.364	C(13)	H(26)[10)]	3.429
C(14)	H(5)[8)]	3.362	C(14)	H(12)	3.263
C(14)	H(12)[11)]	2.774	C(14)	H(13)	2.047
C(14)	H(15)	2.031	C(14)	H(24)[7)]	3.331
C(14)	H(25)[10)]	3.229	C(14)	H(26)[10)]	2.978
C(15)	H(1)[7)]	3.112	C(15)	H(5)[8)]	3.082
C(15)	H(13)	3.269	C(15)	H(14)	2.026
C(15)	H(25)[10)]	3.029	C(16)	H(1)[7)]	3.019
C(16)	H(5)[8)]	2.842	C(16)	H(12)	3.281
C(16)	H(14)	3.263	C(16)	H(15)	2.051
C(16)	H(22)[2)]	2.940	C(16)	H(23)[2)]	3.532
C(18)	H(1)	3.158	C(18)	H(1)[7)]	3.018
C(18)	H(19)	1.983	C(18)	H(20)	2.742
C(18)	H(22)[2)]	3.105	C(18)	H(27)[2)]	3.549
C(19)	H(1)	2.144	C(19)	H(1)[7)]	3.206
C(19)	H(20)	2.083	C(19)	H(22)	3.299
C(19)	H(26)	3.505	C(19)	H(27)	3.206
C(19)	H(27)[2)]	3.532	C(20)	H(1)	3.274
C(20)	H(19)	2.019	C(20)	H(22)	2.686

Tab. S10. (Cont.)

atom	atom	distance	atom	atom	distance
C(20)	H(26)	2.680	C(20)	H(27)	1.990
C(21)	H(1)	3.356	C(21)	H(15)[2)]	3.304
C(21)	H(19)	2.724	C(21)	H(20)	2.047
C(21)	H(22)	2.039	C(21)	H(23)	3.271
C(21)	H(25)	3.279	C(21)	H(25)[12)]	3.333
C(21)	H(26)	2.040	C(21)	H(27)	2.692
C(22)	H(15)[2)]	3.463	C(22)	H(19)	3.078
C(22)	H(20)	3.271	C(22)	H(23)	2.035
C(22)	H(24)	3.268	C(22)	H(25)[12)]	3.153
C(22)	H(26)	3.258	C(22)	H(27)	3.022
C(23)	H(4)[12)]	3.438	C(23)	H(12)[13)]	3.261
C(23)	H(13)[13)]	3.414	C(23)	H(14)[2)]	3.520
C(23)	H(15)[2)]	3.405	C(23)	H(22)	2.034
C(23)	H(24)	2.040	C(23)	H(25)	3.254
C(23)	H(25)[12)]	3.124	C(24)	H(4)[12)]	3.028
C(24)	H(12)[13)]	3.199	C(24)	H(14)[2)]	2.779
C(24)	H(15)[2)]	3.174	C(24)	H(22)	3.266
C(24)	H(23)	2.037	C(24)	H(25)	2.028
C(24)	H(25)[12)]	3.280	C(24)	H(26)	3.256
C(25)	H(14)[9)]	3.257	C(25)	H(14)[2)]	3.125
C(25)	H(15)[9)]	3.272	C(25)	H(15)[2)]	2.974
C(25)	H(23)	3.252	C(25)	H(24)	2.031
C(25)	H(25)[12)]	3.456	C(25)	H(26)	2.036
C(26)	H(4)	3.510	C(26)	H(14)[9)]	2.894
C(26)	H(15)[2)]	3.046	C(26)	H(20)	2.609
C(26)	H(22)	3.259	C(26)	H(24)	3.261
C(26)	H(25)	2.039	C(26)	H(25)[12)]	3.470
H(1)	O(1)	2.411	H(1)	O(3)[2)]	2.809
H(1)	N(17)[2)]	2.122	H(1)	C(2)	1.955
H(1)	C(3)	3.298	H(1)	C(15)[2)]	3.112
H(1)	C(16)[2)]	3.019	H(1)	C(18)	3.158
H(1)	C(18)[2)]	3.018	H(1)	C(19)	2.144
H(1)	C(19)[2)]	3.206	H(1)	C(20)	3.274
H(1)	C(21)	3.356	H(1)	H(15)[2)]	2.567
H(1)	H(19)	2.211	H(1)	H(19)[2)]	2.622
H(1)	H(27)[3)]	3.032	H(1)	H(27)[2)]	3.499
H(4)	O(1)	2.647	H(4)	C(2)	2.609

Tab. S10. (Cont.)

atom	atom	distance	atom	atom	distance
H(4)	C(3)	2.047	H(4)	C(5)	2.026
H(4)	C(6)	3.248	H(4)	C(8)	3.269
H(4)	C(10)[1)]	3.538	H(4)	C(11)[1)]	3.471
H(4)	C(12)[1)]	3.345	H(4)	C(23)[4)]	3.438
H(4)	C(24)[4)]	3.028	H(4)	C(26)	3.510
H(4)	H(5)	2.329	H(4)	H(7)[1)]	3.059
H(4)	H(12)[1)]	3.272	H(4)	H(13)[9)]	3.359
H(4)	H(23)[4)]	3.140	H(4)	H(24)[4)]	2.324
H(4)	H(26)	2.635	H(5)	N(17)[1)]	3.486
H(5)	C(3)	3.284	H(5)	C(4)	2.034
H(5)	C(6)	2.040	H(5)	C(7)	3.262
H(5)	C(11)[1)]	2.927	H(5)	C(12)[1)]	3.227
H(5)	C(13)[9)]	3.229	H(5)	C(13)[1)]	3.429
H(5)	C(14)[1)]	3.362	H(5)	C(15)[1)]	3.082
H(5)	C(16)[1)]	2.842	H(5)	H(4)	2.329
H(5)	H(6)	2.340	H(5)	H(12)[9)]	3.358
H(5)	H(13)[9)]	2.513	H(5)	H(15)[1)]	3.570
H(6)	O(1)[8)]	3.243	H(6)	O(2)[8)]	3.454
H(6)	O(3)[1)]	2.983	H(6)	N(9)[8)]	3.542
H(6)	C(2)[8)]	3.279	H(6)	C(4)	3.249
H(6)	C(5)	2.035	H(6)	C(7)	2.030
H(6)	C(8)	3.266	H(6)	H(5)	2.340
H(6)	H(7)	2.327	H(6)	H(19)[14)]	3.254
H(6)	H(22)[14)]	3.329	H(6)	H(27)[1)]	2.924
H(7)	O(1)[8)]	2.629	H(7)	O(2)	2.470
H(7)	N(9)	2.575	H(7)	C(2)[8)]	2.864
H(7)	C(3)	3.269	H(7)	C(3)[8)]	2.603
H(7)	C(4)[8)]	2.798	H(7)	C(5)	3.260
H(7)	C(5)[8)]	3.439	H(7)	C(6)	2.030
H(7)	C(8)	2.038	H(7)	C(8)[8)]	3.126
H(7)	C(10)	2.608	H(7)	H(4)[8)]	3.059
H(7)	H(6)	2.327	H(7)	H(20)[1)]	3.594
H(7)	H(27)[1)]	3.221	H(12)	O(2)	2.589
H(12)	C(10)	2.638	H(12)	C(11)	2.047
H(12)	C(13)	2.025	H(12)	C(13)[5)]	2.853
H(12)	C(14)	3.263	H(12)	C(14)[5)]	2.774
H(12)	C(16)	3.281	H(12)	C(23)[6)]	3.261

Tab. S10. (Cont.)

atom	atom	distance	atom	atom	distance
H(12)	C(24)[6)]	3.199	H(12)	H(4)[8)]	3.272
H(12)	H(5)[10)]	3.358	H(12)	H(13)	2.325
H(12)	H(13)[5)]	2.728	H(12)	H(14)[5)]	2.591
H(12)	H(23)[6)]	2.837	H(12)	H(23)[2)]	3.147
H(12)	H(24)[6)]	2.725	H(13)	O(2)[11)]	2.843
H(13)	C(4)[10)]	3.367	H(13)	C(5)[10)]	2.907
H(13)	C(10)[11)]	3.471	H(13)	C(11)	3.261
H(13)	C(12)	2.025	H(13)	C(12)[11)]	3.289
H(13)	C(14)	2.047	H(13)	C(15)	3.269
H(13)	C(23)[6)]	3.414	H(13)	H(4)[10)]	3.359
H(13)	H(5)[10)]	2.513	H(13)	H(12)	2.325
H(13)	H(12)[11)]	2.728	H(13)	H(14)	2.345
H(13)	H(23)[6)]	2.626	H(13)	H(26)[10)]	3.432
H(14)	O(2)[11)]	3.194	H(14)	C(12)	3.257
H(14)	C(12)[11)]	3.531	H(14)	C(13)	2.044
H(14)	C(15)	2.026	H(14)	C(16)	3.263
H(14)	C(23)[7)]	3.520	H(14)	C(24)[7)]	2.779
H(14)	C(25)[10)]	3.257	H(14)	C(25)[7)]	3.125
H(14)	C(26)[10)]	2.894	H(14)	H(12)[11)]	2.591
H(14)	H(13)	2.345	H(14)	H(15)	2.328
H(14)	H(24)[7)]	2.583	H(14)	H(25)[10)]	3.272
H(14)	H(25)[7)]	3.169	H(14)	H(26)[10)]	2.624
H(15)	O(1)[7)]	3.168	H(15)	N(1)[7)]	3.045
H(15)	N(17)	2.586	H(15)	C(2)[7)]	3.336
H(15)	C(11)	3.278	H(15)	C(13)	3.273
H(15)	C(14)	2.031	H(15)	C(16)	2.051
H(15)	C(21)[7)]	3.304	H(15)	C(22)[7)]	3.463
H(15)	C(23)[7)]	3.405	H(15)	C(24)[7)]	3.174
H(15)	C(25)[10)]	3.272	H(15)	C(25)[7)]	2.974
H(15)	C(26)[7)]	3.046	H(15)	H(1)[7)]	2.567
H(15)	H(5)[8)]	3.570	H(15)	H(14)	2.328
H(15)	H(25)[10)]	2.939	H(15)	H(25)[7)]	3.354
H(15)	H(26)[7)]	3.468	H(19)	O(3)	2.377
H(19)	O(3)[2)]	2.778	H(19)	N(1)	1.938
H(19)	N(1)[7)]	3.251	H(19)	N(9)	3.223
H(19)	N(17)	2.356	H(19)	C(2)	3.196
H(19)	C(18)	1.983	H(19)	C(20)	2.019

Tab. S10. (Cont.)

atom	atom	distance	atom	atom	distance
H(19)	C(21)	2.724	H(19)	C(22)	3.078
H(19)	H(1)	2.211	H(19)	H(1)[7)]	2.622
H(19)	H(6)[15)]	3.254	H(19)	H(20)	2.785
H(19)	H(22)	2.856	H(19)	H(27)	3.290
H(19)	H(27)[2)]	2.837	H(20)	O(1)[16)]	3.581
H(20)	O(2)[8)]	3.249	H(20)	O(3)	1.963
H(20)	N(1)	2.892	H(20)	N(9)	3.116
H(20)	C(2)	3.152	H(20)	C(3)	2.709
H(20)	C(4)	3.362	H(20)	C(7)	3.493
H(20)	C(8)	2.768	H(20)	C(18)	2.742
H(20)	C(19)	2.083	H(20)	C(21)	2.047
H(20)	C(22)	3.271	H(20)	C(26)	2.609
H(20)	H(7)[8)]	3.594	H(20)	H(19)	2.785
H(20)	H(22)	3.541	H(20)	H(26)	2.440
H(20)	H(27)	2.289	H(22)	O(3)	2.580
H(22)	N(9)[7)]	3.332	H(22)	N(17)[7)]	2.938
H(22)	C(10)[7)]	3.216	H(22)	C(11)[7)]	3.042
H(22)	C(16)[7)]	2.940	H(22)	C(18)[7)]	3.105
H(22)	C(19)	3.299	H(22)	C(20)	2.686
H(22)	C(21)	2.039	H(22)	C(23)	2.034
H(22)	C(24)	3.266	H(22)	C(26)	3.259
H(22)	H(6)[15)]	3.329	H(22)	H(19)	2.856
H(22)	H(20)	3.541	H(22)	H(23)	2.332
H(22)	H(25)[12)]	3.581	H(22)	H(27)	2.794
H(23)	C(10)[7)]	3.575	H(23)	C(11)[7)]	3.018
H(23)	C(12)[13)]	3.162	H(23)	C(12)[7)]	2.932
H(23)	C(13)[13)]	3.053	H(23)	C(13)[7)]	3.364
H(23)	C(16)[7)]	3.532	H(23)	C(21)	3.271
H(23)	C(22)	2.035	H(23)	C(24)	2.037
H(23)	C(25)	3.252	H(23)	H(4)[12)]	3.140
H(23)	H(12)[13)]	2.837	H(23)	H(12)[7)]	3.147
H(23)	H(13)[13)]	2.626	H(23)	H(22)	2.332
H(23)	H(24)	2.341	H(23)	H(25)[12)]	3.537
H(24)	O(1)[12)]	2.754	H(24)	O(2)[13)]	3.571
H(24)	C(2)[12)]	3.523	H(24)	C(4)[12)]	3.229
H(24)	C(12)[13)]	3.402	H(24)	C(14)[2)]	3.331
H(24)	C(22)	3.268	H(24)	C(23)	2.040

Tab. S10. (Cont.)

atom	atom	distance	atom	atom	distance
H(24)	C(25)	2.031	H(24)	C(26)	3.261
H(24)	H(4)[12]	2.324	H(24)	H(12)[13]	2.725
H(24)	H(14)[2]	2.583	H(24)	H(23)	2.341
H(24)	H(25)	2.331	H(24)	H(26)[12]	3.568
H(25)	O(1)[12]	3.361	H(25)	C(14)[9]	3.229
H(25)	C(15)[9]	3.029	H(25)	C(21)	3.279
H(25)	C(21)[4]	3.333	H(25)	C(22)[4]	3.153
H(25)	C(23)	3.254	H(25)	C(23)[4]	3.124
H(25)	C(24)	2.028	H(25)	C(24)[4]	3.280
H(25)	C(25)[4]	3.456	H(25)	C(26)	2.039
H(25)	C(26)[4]	3.470	H(25)	H(14)[9]	3.272
H(25)	H(14)[2]	3.169	H(25)	H(15)[9]	2.939
H(25)	H(15)[2]	3.354	H(25)	H(22)[4]	3.581
H(25)	H(23)[4]	3.537	H(25)	H(24)	2.331
H(25)	H(26)	2.335	H(26)	O(1)	3.559
H(26)	N(1)	3.301	H(26)	C(2)	3.018
H(26)	C(3)	2.982	H(26)	C(4)	2.849
H(26)	C(13)[9]	3.429	H(26)	C(14)[9]	2.978
H(26)	C(19)	3.505	H(26)	C(20)	2.680
H(26)	C(21)	2.040	H(26)	C(22)	3.258
H(26)	C(24)	3.256	H(26)	C(25)	2.036
H(26)	H(4)	2.635	H(26)	H(13)[9]	3.432
H(26)	H(14)[9]	2.624	H(26)	H(15)[2]	3.468
H(26)	H(20)	2.440	H(26)	H(24)[4]	3.568
H(26)	H(25)	2.335	H(27)	O(1)[16]	1.871
H(27)	N(1)[16]	3.372	H(27)	N(17)[7]	3.024
H(27)	C(2)[16]	2.829	H(27)	C(6)[8]	3.491
H(27)	C(18)[7]	3.549	H(27)	C(19)	3.206
H(27)	C(19)[7]	3.532	H(27)	C(20)	1.990
H(27)	C(21)	2.692	H(27)	C(22)	3.022
H(27)	H(1)[16]	3.032	H(27)	H(1)[7]	3.499
H(27)	H(6)[8]	2.924	H(27)	H(7)[8]	3.221
H(27)	H(19)	3.290	H(27)	H(19)[7]	2.837
H(27)	H(20)	2.289	H(27)	H(22)	2.794

Symmetry Operators:

(1) -X+1,Y+1/2-1,-Z+1
(2) -X+2,Y+1/2-1,-Z+1
(3) X,Y-1,Z
(4) -X+2,Y+1/2-1,-Z+2
(5) -X+1,Y+1/2-1,-Z
(6) X-1,Y,Z-1
(7) -X+2,Y+1/2,-Z+1
(8) -X+1,Y+1/2,-Z+1
(9) X,Y,Z+1
(10) X,Y,Z-1
(11) -X+1,Y+1/2,-Z
(12) -X+2,Y+1/2,-Z+2
(13) X+1,Y,Z+1
(14) X-1,Y,Z
(15) X+1,Y,Z
(16) X,Y+1,Z

References

[27] Altomare A, Cascarano G, Giacovazzo C, Guagliardi A, Burla M, Polidori G, Camalli M.
SIRPOW.92 - a program for automatic solution of crystal structures by direct methods optimized for powder data.
J Appl Cryst. 1994; 27: 435–436.
http://dx.doi.org/10.1107/S0021889894000221

[28] DIRDIF99: Beurskens, P.T., Admiraal, G., Beurskens, G., Bosman, W.P., de Gelder, R., Israel, R. and Smits, J.M.M.(1999). The DIRDIF-99 program system, Technical Report of the Crystallography Laboratory, University of Nijmegen, The Netherlands.

[29] Least Squares function minimized:
$\Sigma w(F_O^2 - F_c^2)^2$ where
w = Least Squares weights.

[30] Standard deviation of an observation of unit weight:
$[\Sigma w(F_O^2 - F_c^2)^2/(N_O - N_v)]^{1/2}$
where N_O = number of observations
N_V = number of variables.

[31] Flack HD.
On enantiomorph-polarity estimation
Acta Cryst. 1983; A39: 876–881.
http://dx.doi.org/10.1107/S0108767383001762

[32] Cromer DT, Waber JT.
International Tables for X-ray Crystallography.
Vol. IV, The Kynoch Press, Birmingham, England, Table 2.2 A (1974).

[33] Ibers JA, Hamilton WC.
Dispersion corrections and crystal structure refinements.
Acta Crystallogr. 1964; 17: 781–782.
http://dx.doi.org/10.1107/S0365110X64002067

[34] Creagh DC, McAuley WJ.
International Tables for Crystallography.
Vol C, (Wilson AJC, ed.), Kluwer Academic Publishers, Boston, Table 4.2.6.8, pages 219–222 (1992).

[35] Creagh DC, Hubbell JH.
International Tables for Crystallography.
Vol C, (Wilson AJC, ed.), Kluwer Academic Publishers, Boston, Table 4.2.4.3, pages 200–206 (1992).

[36] CrystalStructure 3.8: Crystal Structure Analysis Package, Rigaku and Rigaku Americas (2000–2007). 9009 New Trails Dr. The Woodlands TX 77381 USA.

[37] Carruthers JR, Rollett JS, Betteridge PW, Kinna D, Pearce L, Larsen A, Gabe E.
CRYSTALS Issue 11:
Chemical Crystallography Laboratory, Oxford, UK. (1999).
